# Electrolyte Anions Suppress Hydrogen Generation in Electrochemical CO Reduction on Cu

**DOI:** 10.1002/anie.202421196

**Published:** 2025-01-10

**Authors:** Lee Fuller, Gong Zhang, Seonmyeong Noh, Reid C. Van Lehn, Marcel Schreier

**Affiliations:** ^1^ Department of Chemical and Biological Engineering University of Wisconsin-Madison Madison WI 53706 United States; ^2^ Department of Chemistry University of Wisconsin-Madison Madison WI 53706 United States

**Keywords:** CO_2_ reduction, Electrochemistry, Electrocatalysis, Anions, Heterogeneous catalysis

## Abstract

In this study, we employed electrochemical‐mass spectrometry (EC‐MS) to elucidate the role of halide anions in electrochemical CO_2_ and CO reduction. We found that the undesired hydrogen evolution reaction (HER) was significantly suppressed by the anion used. Specifically, the rates of H_2_ production decreased in the order KF > KCl > KI, meaning that I^−^ most strongly suppressed HER. Interestingly, CO reduction products showed an inverse relationship to HER, with KI leading to the highest rate of CO reduction. By pairing our experimental findings with classical molecular dynamics simulations, we propose a mechanism wherein halide anions influence the dynamic interplay between CO reduction and HER by modulating the competition of H* and CO* for active sites on the Cu surface. We propose that this interaction is enabled by the interfacial concentration of K^+^ being greater in the presence of F^−^ than in I^−^. Our results highlight the need to more broadly consider the properties of ions at electrocatalytic interfaces and they point to thus far underappreciated avenues to optimize hydrocarbon production while suppressing hydrogen evolution.

## Graphical abstract

We studied the role of halide anions in Cu catalyzed electrochemical CO reduction (eCOR). Our findings show that anions suppress the undesired hydrogen evolution reaction (HER). By pairing our experimental results with classical molecular dynamics (MD) simulations, we propose a model wherein anions modulate the interplay between adsorbed CO* and H* at the catalyst surface, controlling the outcome of the CO reduction reaction.

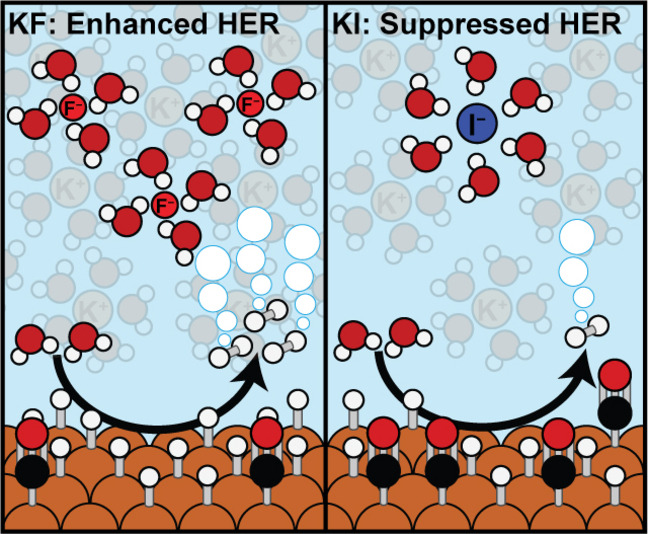

## Introduction

The electrochemical reduction of CO_2_ (eCO_2_R) promises novel avenues for storing renewable electricity and for the sustainable production of feedstock chemicals.[^1^, ^2^] Copper has long stood out as a unique electrocatalyst for this reaction because it catalyzes the transformation of CO_2_ into valuable hydrocarbons and oxygenates, such as methane and ethylene, among many others.[^1^, ^3^] However, despite extensive research efforts, the reaction continues to suffer from high overpotentials, poor product selectivity, and competition with the hydrogen evolution reaction (HER).^[2]^ To understand the origin of these challenges and to develop avenues to address them, substantial efforts have been dedicated to understand the mechanism of Cu‐catalyzed CO_2_ reduction. These investigations have led to the realization that the electrolyte has a major impact on the reaction rate and selectivity of eCO_2_R.[^4^, ^5^, ^6^, ^7^, ^8^] In particular, electrolyte cations have been shown to modulate the formation of C_2+_ products, putatively by interacting with the electron‐transfer transitions states leading to the formation of these products.[^9^, ^10^, ^11^, ^12^, ^13^, ^14^, ^15^, ^16^, ^17^, ^18^] Yet, numerous questions remain unaddressed. In particular, the role of anions in eCO_2_R is less clear and discussion of their role has primarily focused on their activity as pH buffers,[^19^, ^20^, ^21^] and their supposed adsorption to electrode surfaces.[^22^, ^23^, ^24^, ^25^, ^26^, ^27^, ^28^, ^29^, ^30^, ^31^] In addition, questions remain as to how CO_2_ reduction and hydrogen evolution intersect and how the properties of the electrochemical interface control each process. This lack of insight may partially be explained by shortcomings in the experimental methodologies used to investigate CO_2_ reduction reactions. Typically, steady‐state electrolysis is performed over long periods of time. However, analysis of these long experiments is convoluted by several phenomena such as deposition of carbonates, catalyst reconstruction and degradation, and mass transport limitations.^[32]^

**Figure 1:**
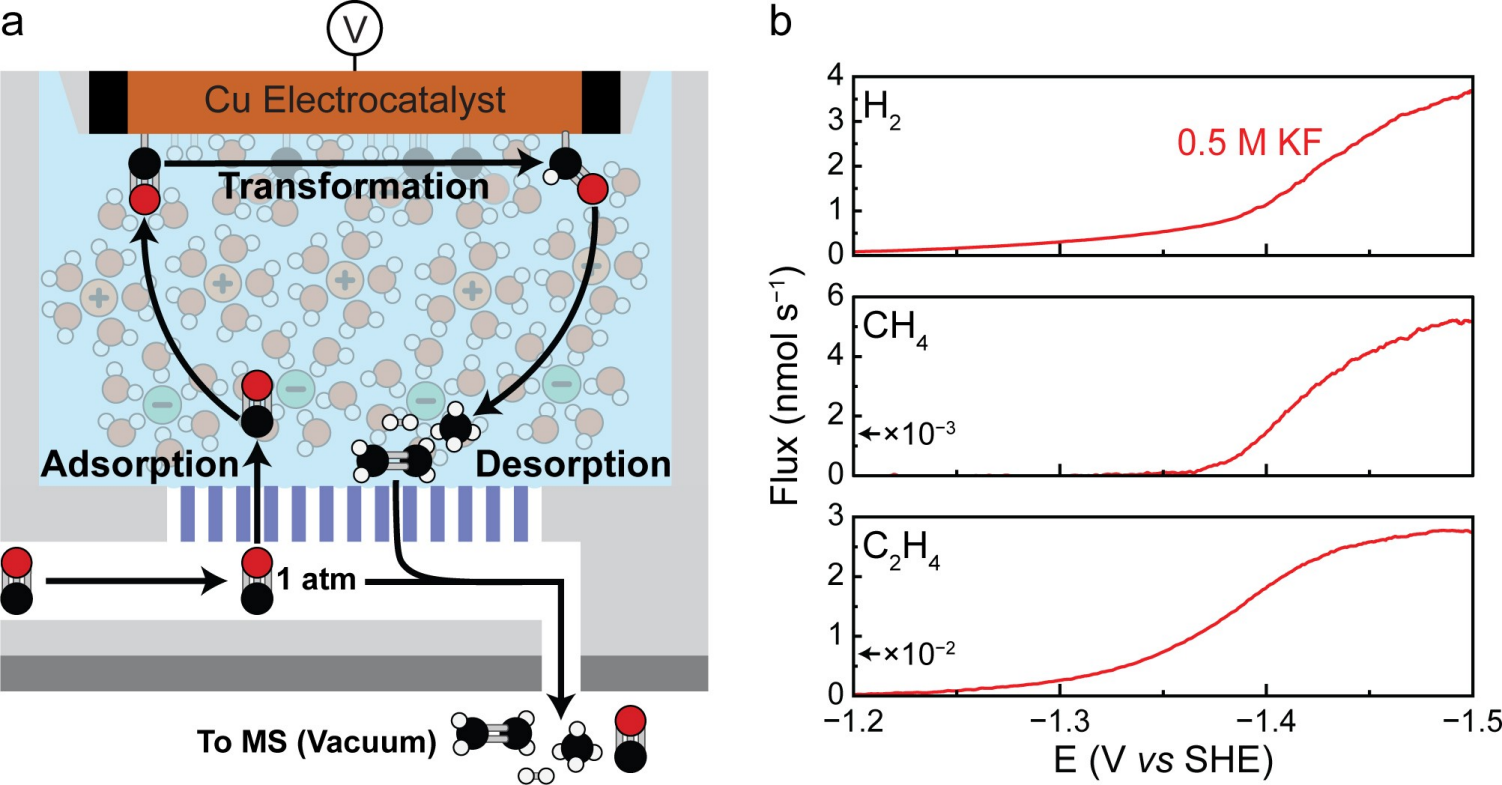
(a) Electrochemical‐mass spectrometry (EC‐MS) allows for real time product analysis, and thus gives insights into transient phenomena driving electrocatalysis. (b) Linear sweep voltammetry (LSV) was performed at a scan rate of 1 mV s^−1^ in 0.5 M KF under CO. EC‐MS analysis shows the production rate of H_2_, CH_4_, and C_2_H_4_. The presented data is the average of three independent measurements. Replicates are shown in Figure S6.

To alleviate some of the issues associated with steady‐state electrolysis, we herein used electrochemical‐mass spectrometry (EC‐MS, Figure 1a, Figure S1‐Figure S5) to study the dynamic evolution of products on Cu during the electrocatalytic reduction of CO and to understand how these processes are influenced by anion identity. EC‐MS brings a mass spectrometer into close contact with an electrochemical interface,^[33]^ allowing for rapid product analysis during the course of potential sweeps.

Our data provide insight into the dynamics among H* and CO* adsorbates in generating hydrocarbon products and undesired hydrogen. They further show how the nature of electrolyte anions modulates interfacial properties influencing HER, demonstrating that anions can be used to suppress H_2_ production. Our findings help bridge the gaps between previously proposed roles of anions in electrochemical CO_2_ and CO reduction.

## Results and Discussion

### Baseline: CO Reduction in the Presence of F^−^

To establish a baseline for the product distribution observed from electrochemical CO reduction (eCOR) on copper, we carried out eCOR in the presence of 0.5 M KF. We used CO as substrate to avoid convolution of our data by the complex speciation of CO_2_ in aqueous solutions.^[2]^ F^−^ anions were chosen as a baseline for comparing the behavior of other anions since F^−^ is reported to only weakly interact with Cu.[^26^, ^27^] In all experiments, glassy carbon was used as a conductive support for copper nanoparticles (25 nm diameter), which served as the working electrode in an aqueous electrolyte. EC‐MS was used to quantify the generation of products in real‐time during linear potential sweeps. A detailed description of the experimental protocol can be found in the Supporting Information.

Linear sweep voltammetry (LSV) was performed on three independent samples at 1 mV s^−1^ from −0.88 V to −1.5 V (all potentials reported *vs* SHE) in 0.5 M KF, resulting in the averaged product distribution shown in Figure 1b (see Figure S6 for replicates). At low overpotentials, only hydrogen generation was observed. As the overpotential increased, we started to see the production of ethylene at around −1.20 V, followed by methane at −1.37 V. The observed Tafel slopes for both ethylene and methane production agreed with previous reports. Ethylene featured a Tafel slope of ~112 mV dec^−1^ (Table S1), which has been explained by a mechanism where two *CO adsorbates take up an electron to form a C−C bond, resulting in the formation of a CO dimer (*OCCO^−^).[^34^, ^35^, ^36^] The production of methane, on the other hand, displayed a Tafel slope of ~39 mV dec^−1^ (Table S2), which might be representative of a mechanism involving a pre‐equilibrated electron transfer followed by a rate‐limiting electron transfer.[^35^, ^37^] At −1.37 V, in concert with the onset of methane production, the rate of hydrogen generation increased substantially. This observation may be ascribed to the transfer of hydrogen atoms that are involved in both methane and H_2_ formation.^[36]^ Interestingly, at strongly reductive potentials, the production of hydrogen, methane, and ethylene all reached a plateau and became potential‐independent. The appearance of this plateau was nearly independent of the potential sweep rate (Figure S7). We hypothesized that the plateau may originate from the saturation of the catalyst surface with adsorbed species. However, the generation of deposits such as saturated KOH cannot be ruled out.

### HER Mechanism Changes in the Presence of CO

**Figure 2:**
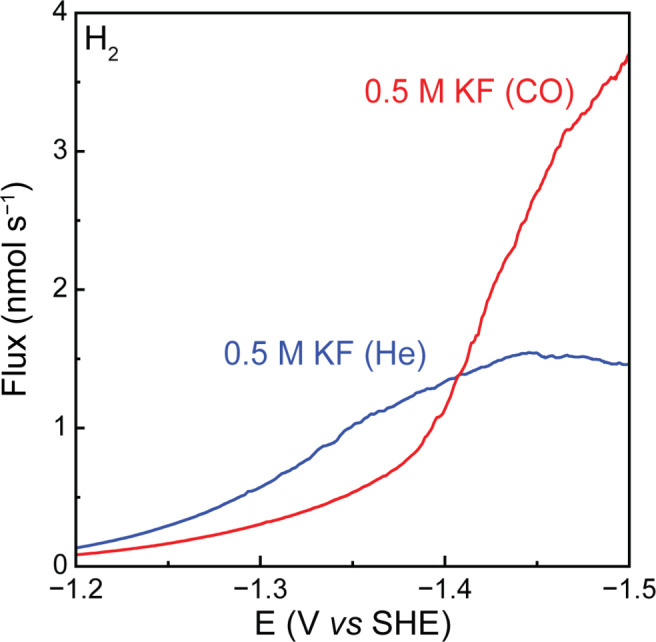
Linear sweep voltammetry (LSV) was performed at a scan rate of 1 mV s^−1^ in 0.5 M KF under CO and under He. EC‐MS analysis shows the production rates of H_2_ in the presence of CO and He. The presented data is the average of three independent measurements. Replicates are shown in Figure S6.

Comparing the potential‐dependent HER rate under He with the HER rate under CO shows that while CO suppresses HER at low potentials, the presence of CO leads to a *higher rate of HER* at high driving forces. It has commonly been reported that in the presence of CO, fewer surface sites are available for hydrogen atoms to adsorb and to form H_2_, leading to a lower HER rate.[^38^, ^39^, ^40^] We indeed observed this suppression at potentials positive of −1.4 V (Figure 2). However, at potentials negative of −1.4 V, the rate of hydrogen evolution under CO exceeds the HER rate under He. This coincided with the onset of CH_4_ production (Figure S8a) and with a lowering of the Tafel slope for HER in the presence of CO (Figure S8b). We theorize that this observation may point to a change in the dominant HER mechanism.

Under normal conditions, i.e. in presence of He, HER on Cu has been suggested to take place through a Volmer–Tafel mechanism.^[41]^ Introducing CO* is known to lower the hydrogen affinity of surfaces.[^38^, ^42^] Forming adsorbed H* atoms in the presence of CO* thus requires more negative potentials but also creates more labile H* adsorbates, for which, rather than binding a second H* with the same energy cost, coupled transfer of a hydrogen atom from H_2_O and an electron to an adsorbed H* atom could become more favorable, resulting in a Heyrovsky‐type mechanism (Figure S9). This might explain the decrease in Tafel slope, as shown in Figure S8b. It also agrees with a prior literature report that indicated a transition from a Volmer–Tafel to a Volmer–Heyrovsky mechanism for HER on Cu when benzaldehyde was adsorbed on the surface.^[41]^ The observed *increase* in HER rate in the presence of CO is unexpected and has important implications for achieving high Faradaic efficiencies of CO_2_ reduction products.

### Impact of Anion Identity on eCOR and HER

We found that the interaction between CO reduction and HER is significantly modulated by the identity of the anions present in the electrolyte. To gain insight, we used EC‐MS to generate high resolution rate data for eCOR product formation in the presence of a series of halide anions. During all experiments, the total cation concentration was kept constant at 0.5 M K^+^. This was achieved by using 0.25 M KF as a supporting electrolyte and adjusting the concentration of the anion potassium salt to yield a total cation concentration of 0.5 M K^+^. In addition to 0.5 M KF, the electrolytes tested were 0.25 M KF+0.25 M KCl and 0.25 M KF+0.25 M KI. Here, the 0.5 M KF electrolyte will be referred to as ‘F^−^’, while the other electrolytes will be referred to by the other anion present in the mixture.

**Figure 3:**
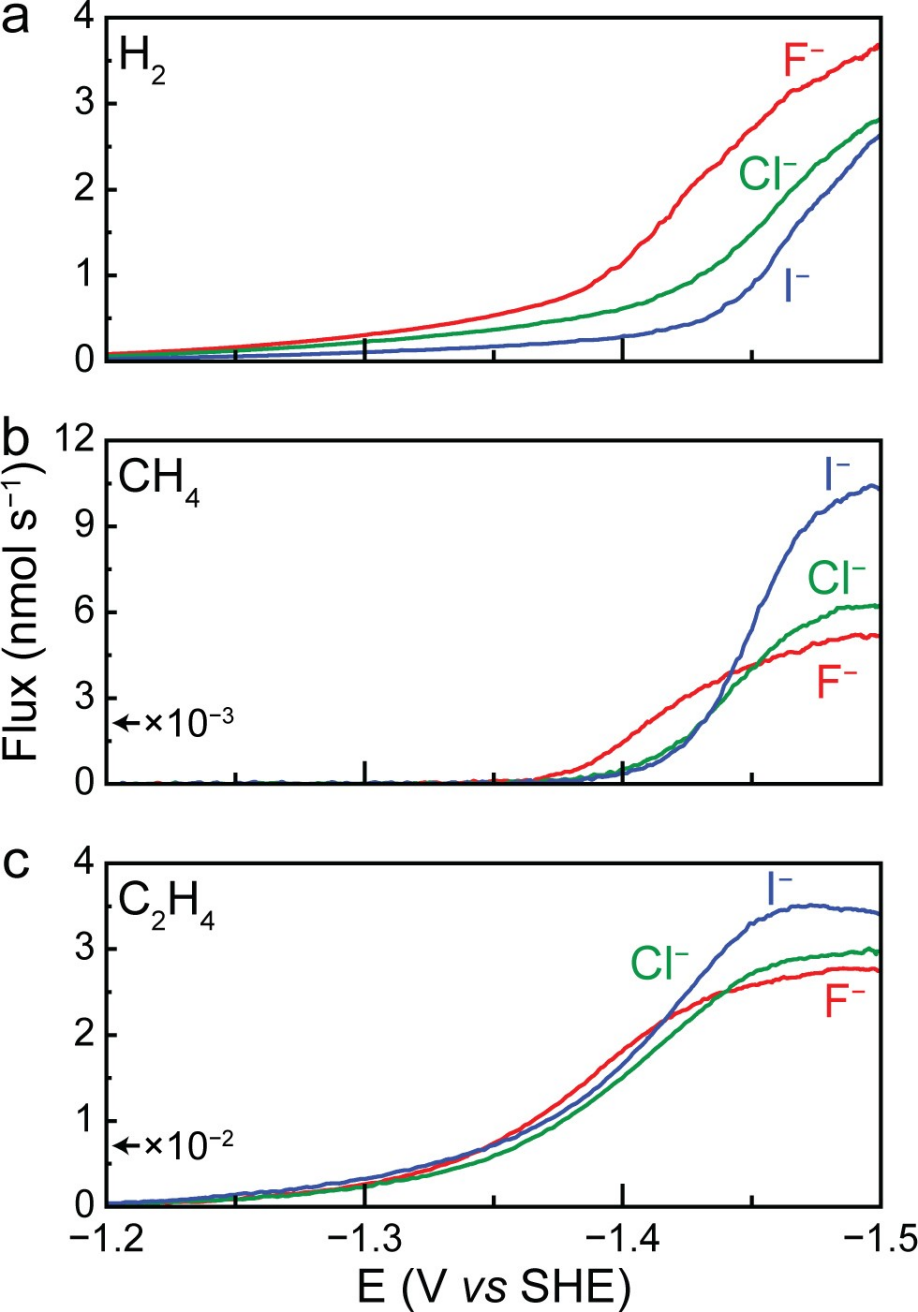
Linear sweep voltammetry (LSV) was performed at a scan rate of 1 mV s^−1^ under CO in the following electrolytes: 0.5 M KF, 0.25 M KF+0.25 M KCl and 0.25 M KF+0.25 M KI. EC‐MS analysis shows the production rates of (a) H_2_, (b) CH_4_, and (c) C_2_H_4_. The presented data is the average of three independent measurements. Replicates are shown in Figure S6, S10, and S11.

Figure 3a, b, and c show the production of hydrogen, methane and ethylene under CO in the presence of F^−^, Cl^−^, and I^−^. Since eCOR occurs at strongly negative potentials where negatively charged ions are displaced from the electrochemical interface, one could assume that anions minimally influence catalysis.^[43]^ Indeed, ethylene production is minimally impacted by the identity of the electrolyte anion present (Figure 3c). However, in contrast to the relatively small impact on ethylene formation, the electrolyte anion significantly impacts the production of hydrogen and methane (Figure 3a,b). Specifically, Cl^−^ and I^−^ show substantial suppression of hydrogen evolution activity relative to F^−^ (Figure 3a). On the other hand, halide anions that lead to a suppression of HER, simultaneously lead to a relative increase in the rate of CH_4_ production at high overpotentials (Figure 3b).

A possible explanation for these observations would be changes in the electrolyte pH. Yet, the poor overlap in Figure S12, which displays hydrogen production for the various electrolytes as a function of RHE calculated from the simulated local pH, points against pH from being responsible for differences in hydrogen production. Similarly, Figure S13 shows differences in methane production on the local pH corrected RHE scale between the various electrolytes, and the poor overlap also points against pH from being the sole factor in explaining differences in CH_4_ production.

Other than the pH, an explanation for the observed anion‐dependence would be the adsorption of anions.[^44^, ^45^, ^46^] Within the literature, there are multiple hypotheses on how specifically adsorbed halides could influence eCO_2_R/eCOR. Some reports have suggested that specifically adsorbed anions could donate charge to CO* or CO_2_ and thereby strengthen their adsorption to Cu.[^28^, ^47^] Other studies claimed that adsorbed halides indirectly strengthen the adsorption of CO* by modifying the electronic structure of Cu sites.[^29^, ^30^] Yet, direct evidence for anion specific adsorption under CO_2_ reduction potentials is lacking, and some reports mention explicitly that they could not detect the presence of adsorbed iodide species under experimental conditions using in situ Raman spectroscopy.[^28^, ^30^] In addition, previous literature reports indicate that on Cu, at −0.913 V *vs* SHE the surface coverage of Cl^−^ becomes negligible, pointing against an important role of specific anion adsorption.^[48]^ Indeed, when we performed classical molecular dynamics (MD) simulations of the interface formed between Cu(100) and electrolytes containing 0.5 M KF and 0.5 M KI, we found that that neither F^−^ or I^−^ specifically adsorb to Cu(100) under the simulated conditions. The MD simulations were performed using a constant charge method (calibrated as discussed in the Supporting Information) in which the charge densities at the electrodes were set such that the negative electrode was at −0.7 V *vs* potential of zero charge (PZC), which we estimate to correspond to −1.43 V *vs* SHE based on a PZC of Cu of −0.73 V *vs* SHE.[^49^, ^50^]

Combined with our experimental results, our MD simulations and prior literature suggests that possibly something other than the specific adsorption of anions may be leading to the beneficial impact of anions on HER suppression and CH_4_ production.

**Figure 4:**
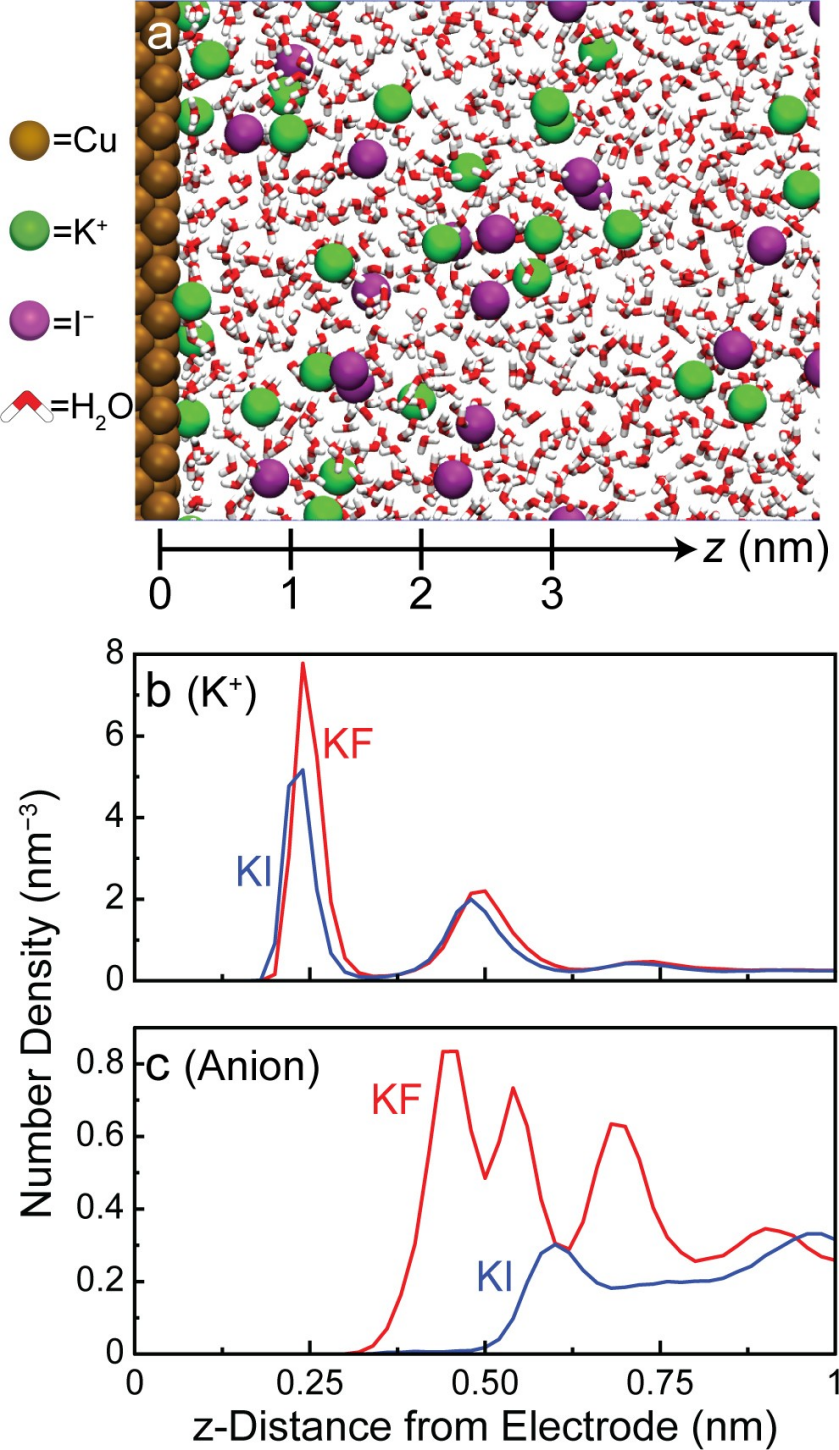
Classical molecular dynamics (MD) simulations were performed to model 0.5 M KF and 0.5 M KI electrolytes confined between Cu(100) electrodes at −0.7 V *vs* PZC. (a) Snapshot of the 0.5 M KI simulation system near the negatively charged electrode. The *z*‐axis is drawn at the bottom with the value of *z*=0 nm indicated for comparison to Figure S29. Some water molecules are removed for visual clarity. Cu atoms are brown, K^+^ ions are green, I^−^ ions are purple, and the red and white molecules are water (oxygen atom is represented by red and the hydrogen atoms are represented by white). (b) Local K^+^ concentration and (c) local anion concentration near the negatively charged electrode in 0.5 M KF and 0.5 M KI.

We suggest that our observations may instead be explained by an interplay between electrolyte anions and cations, which modulates the supply of H* to the electrode surface and thereby controls the surface reactions leading to HER, CH_4_, and C_2_H_4_ production. It has been suggested that the identity and concentration of electrolyte cations play a critical role in determining the rate of HER in neutral to alkaline electrolytes.[^51^, ^52^, ^53^] The reason for this is that electrolyte cations are needed to stabilize the transition state leading to the formation of surface‐H* species (Figure S14).^[51]^ Here, we suggest that these cation‐induced effects may be modulated by the anions present in the electrolyte. Specifically, our MD simulations of Cu(100) in 0.5 M KF and 0.5 M KI at −0.7 V *vs* PZC (Figure 4a) indicate that the chemical identity of the anion present in the electrolyte controls the effective concentration of cations near the electrode surface (Figure 4b). This may be because the positive charge on the cation needs to be partially compensated by anions for cations to be able to exist at high concentration at a point in space.[^54^, ^55^] As a result, the concentration of K^+^ in the electrochemical double layer (EDL) is lower in the presence of I^−^ than in the presence of F^−^, likely because the approach of I^−^ to the electrode is hindered by its size, solvation and coulombic interactions with other electrolyte components (Figure 4c). Indeed, our calculations indicate that I^−^ has a larger solvated radius than F^−^ (see SI).[^56^, ^57^] We hypothesize that it is this anion‐mediated modulation of the local cation concentration that influences the rate of water dissociation and thus of HER (under neutral to alkaline conditions, like here). This effect may explain why H_2_ production is enhanced in the presence of F^−^ relative to I^−^ in both CO (Figure 3a) and He (Figure S15) atmosphere.

Recent studies have suggested that cations of different identities can disrupt the hydrogen bonding network of interfacial water and impact eCO_2_R/eCOR and HER rates.[^58^, ^59^] Since the cation identity stays constant in our work, we expect that the interfacial solvation environment remains similar,[^58^, ^59^] suggesting that our data may not be dominated by an effect involving the disruption of interfacial water. Beyond influencing HER, we propose that the anion‐mediated modulation of the rate of water dissociation also controls the outcome of CO reduction reactions. As the rate of water dissociation is increased under greater local concentrations of electrolyte cations (i.e. in F^−^ relative to I^−^, Figure 4b), we expect to observe a greater rate of hydrogen evolution. Indeed, the rate of H_2_ production is greater in F^−^ relative to I^−^ in both CO and He atmospheres (Figure 3a, Figure S15). When water dissociation occurs more readily (i.e. in F^−^), the surface coverage of H* will likely be greater as compared to when water dissociation occurs more slowly. In this case, the surface coverage of CO* relative to H* may decrease since H* and CO* compete for surface sites on Cu.[^6^, ^38^, ^39^, ^40^, ^42^, ^60^, ^61^, ^62^] Thus, fresh hydrogen atoms from water molecules will have a greater likelihood of reacting with H* rather than CO* (relative to when water dissociation occurs more slowly), leading to an increase in H_2_ production relative to CH_4_ production. In contrast, when water dissociation occurs more slowly (i.e. in I^−^), the surface coverage of H* will decrease (relative to when water dissociation is facile), and the surface coverage of CO* relative to H* will increase. Consequently, fresh hydrogen atoms from water molecules will have a higher likelihood of reacting with CO and forming CH_4_ (relative to when water dissociation occurs more rapidly). It is through this mechanism that the rate of water dissociation modulates the relative surface population of H* and CO* at the surface. This mechanism may explain the decreased CH_4_ production rate in the presence of F^−^ relative to I^−^. Similarly, at higher CO coverage (i.e. under I^−^), we expect to observe a higher rate of ethylene formation, which decreases as the CO coverage becomes lower (i.e. under F^−^). This trend can be observed throughout all the halide anions tested herein, whereas the CO reduction rate to methane and ethylene trends inversely with HER rates. Indeed, previous literature supports that relative to other anions, I^−^ led to the greatest coverage of CO* on Cu surfaces.^[30]^

It is important to note that Figure 4b shows that the interfacial cation concentration is greater in the presence of F^−^ than in I^−^. To understand if different local cation concentrations influence the strength of the electric fields at the electrode surface, we calculated the electric fields in KF and KI electrolytes. As shown in Figure S31, the electric field strengths are found to be nearly identical in KF and KI electrolytes. This is because the electric field is largely dominated by the solvent dipoles (H_2_O), as has been described by the Bockris‐Devanathan‐Müller (BDM) model.^[63]^ Our experimental results may thus not be explained by differences in the electric field strengths, but instead by the effective concentration of the ions present at the interface. Furthermore, if the electrostatic field strength was greater in the presence of F^−^ than in I^−^, one could expect that F^−^ would lead to the greatest production rate of ethylene since it has been proposed that the interfacial electric field can impact the stability of the OCCO^−^ intermediate and the resulting production of ethylene.[^13^, ^17^, ^18^] However, Figure 3c shows that at high overpotential, the production rate of ethylene is the lowest in F^−^ and the greatest in I^−^. This discrepancy may also suggest that something other than differences in the electric field strength is responsible for the trends in our data.

Importantly, we continue to observe the anion‐induced suppression of HER between F^−^ and I^−^ in constant‐potential experiments carried out in a gas diffusion electrode (GDE) cell at −1.1, −1.3, and −1.5 V (Figure S16–18). This supports our EC‐MS based findings. In addition, our GDE tests also confirmed the increase in C_2+_ compounds under I^−^ compared to F^−^, which we ascribe to the surface population of CO, as mentioned above. As part of these experiments, we also identified the formation of liquid products at steady‐state. Liquid C_2+_ product formation increased from F^−^ to I^−^ at −1.3 V, in agreement with the hypothesized CO coverage. It is important to note that for steady‐state polarization experiments, mass transport limitations of CO and hydrogen atoms are likely to occur, which may convolute the data.

To support our hypothesis of the role of anions, we need to further consider alternative explanations for the observed phenomena. Halide anions have been suggested to reconstruct Cu surfaces under electrochemical potentials.[^24^, ^25^, ^30^, ^64^] To ensure that the findings mentioned above are not convoluted by anion‐induced surface reconstructions, we performed an electrolyte exchange experiment where the Cu catalyst was first put into contact with 0.25 M KF+0.25 M KI electrolyte, followed by replacement with 0.5 M KF electrolyte (see Figure S19a for experimental details). Throughout the duration of this experiment, the production rate of hydrogen, methane, and ethylene was measured using EC‐MS. If our experimental results were controlled by anion‐induced surface reconstruction, we would expect that the high production rates for hydrocarbons would persist after removing I^−^. However, as shown in Figure S19b, we see that once the KI‐based electrolyte is fully replaced by the pure KF electrolyte, the production of hydrocarbons decreased significantly, while hydrogen increased. These results match the trends observed during our LSV measurements (Figure 3). Our findings thus support prior literature that pointed against anion‐induced surface reconstructions from being the sole contributor to the anion effects observed in CO_2_ reduction.^[30]^ In addition, our MD simulations do not support an interaction of the anions with the electrode surface (Figure 4c), which also points against anion‐induced surface reconstruction under the simulated conditions. Furthermore, to confirm that our results are not affected by halogen evolution at the counter electrode, we performed LSV experiments under CO in 0.25 M KF+0.25 M KI electrolyte with a membrane to prevent product diffusion from the counter to the working electrode. As shown in Figure S20, the production rates of hydrogen, methane, and ethylene are nearly identical with and without the use of a membrane, confirming that our analysis is not convoluted by halogen evolution. Finally, we note that the formation of OH^−^ could modulate the interactions between halides and electrolyte cations.^[65]^ These interactions should be the subject of future studies.

## Conclusion

Herein, we used EC‐MS to provide novel insight into the role of halide anions in electrochemical CO_2_ and CO reduction. We found that electrolyte anions could substantially suppress undesired H_2_ production, while showing an inverse relationship between the rate of CO reduction and H_2_ generation. Notably, I^−^ strongly suppressed H_2_ production and led to greater production rates towards methane and ethylene compared to other halide anions. We hypothesize that the inverse trends between H_2_ production and eCOR production rates may indicate that the electrolyte anion modulates the relative surface coverages of H* and CO*. To further probe the role of anions, we performed classical MD simulations of KF and KI electrolytes at an applied potential relevant to eCOR. We found that under the simulated conditions, F^−^ and I^−^ do not specifically adsorb to Cu. Furthermore, our MD simulations showed that the interfacial concentration of K^+^ near the Cu surface is greater in the presence of F^−^ than in I^−^. Based on this, we propose that non‐specifically adsorbed anions may influence the effective concentration of K^+^ near the Cu surface, which in turn may influence the surface competition between CO* and H*, possibly explaining how anions influence eCOR and HER. Strengthening this hypothesis will require future investigation to directly connect interfacial structure to catalytic outcome. Our experimental results indicate that more consideration should be put into how the properties of non‐specifically adsorbed ions can impact reactions at the electrode surface. Our data also point to important interactions between cations and anions in the electrochemical double layer, which should be the subject of future studies. Overall, our work opens the door to leveraging thus far unappreciated electrolyte effects in eCOR. These effects are important as they allow us to suppress the parasitic HER and increase the production of desirable hydrocarbons.

## Supporting Information

The authors have cited additional references within the Supporting Information.[^1^, ^13^, ^33^, ^34^, ^35^, ^37^, ^41^, ^49^, ^50^, ^51^, ^56^, ^66^, ^67^, ^68^, ^69^, ^70^, ^71^, ^72^, ^73^, ^74^, ^75^, ^76^, ^77^, ^78^, ^79^, ^80^, ^81^, ^82^, ^83^, ^84^, ^85^, ^86^]

## Conflict of Interests

The authors declare no conflict of interest.

1

## Supporting information

As a service to our authors and readers, this journal provides supporting information supplied by the authors. Such materials are peer reviewed and may be re‐organized for online delivery, but are not copy‐edited or typeset. Technical support issues arising from supporting information (other than missing files) should be addressed to the authors.

Supporting Information

Supporting Information

## Data Availability

The data that support the findings of this study are available from the corresponding author upon reasonable request.
