# Supplementary material for: Electrolyte Anions Suppress Hydrogen Generation in Electrochemical CO Reduction on Cu
Source: Angew Chem Int Ed Engl. 2025 Jan 10;64(10):e202421196. doi: 10.1002/anie.202421196 (PMC11878348; doi:10.1002/anie.202421196)
Supplement: Supplementary file 1 — Supporting Information [file ANIE-64-e202421196-s001.pdf]

Angewandte Chemie
Eine Zeitschrift der Gesellschaft Deutscher Chemiker

Supporting Information

Electrolyte Anions Suppress Hydrogen Generation in Electrochemical CO Reduction on Cu

*L. Fuller, G. Zhang, S. Noh, R. C. Van Lehn, M. Schreier**

Supporting Information

Electrolyte Anions Suppress Hydrogen Generation in Electrochemical CO Reduction on Cu

Lee Fuller,[b] Dr. Gong Zhang,[a] Seonmyeong Noh,[a] Prof. Reid C. Van Lehn,[a][b] and Prof. Marcel Schreier*[a][b]

[a] Department of Chemical and Biological Engineering, University of Wisconsin-Madison, Madison, Wisconsin 53706, United States
[b] Department of Chemistry, University of Wisconsin-Madison, Madison, Wisconsin 53706, United States

*Correspondence: mschreier2@wisc.edu

Table of Contents

1. Electrochemical Mass Spectrometry Experimental Procedures S2–S7
    1.1 Materials
    1.2 Calibration of in-situ Electrochemical Mass Spectrometry
    1.3 Voltammetric Experiments
2. Steady-state Electrolysis Experimental Procedures S7–S9
    2.1 Materials
    2.2 Gas Product Analysis
    2.3 Liquid Product Analysis
3. Calculation of Local pH S10
4. Supplemental Figures and Tables S11–S32
5. Molecular Dynamics Simulations S33–S45
6. References S46–S47

1. Electrochemical Mass Spectrometry Experimental Procedures

1.1 Materials

Electrolyte

A Milli-Q Reference A+ System (MilliporeSigma) was used to purify all water used in this study (cleaning, electrolyte preparation, etc.) to give a resistivity of 18.2 MΩ cm. Before use, all potassium-based electrolytes were purified by treatment with Chelex 100 Chelexing Resin Sodium Form (Bio-Rad). The purification procedure was performed as follows:

1. Na^+ ions were removed from Chelex by making a mixture containing a concentration of 0.1 g Chelex/mL of 1 M HCl (ACS Reagent Grade, 37%). This mixture was stirred for 12 hours.
2. The mixture was then filtered and washed with 5 L of Milli-Q water.
3. The filtered Chelex was then converted to the K^+ form by placing it in a mixture containing 0.1 g Chelex/mL of 1 M KOH (Sigma-Aldrich, Semiconductor grade 99.99%). This mixture was stirred for 24 hours on a stir plate set at a surface temperature of 60°C.
4. The mixture was then filtered and washed with 8 L of Milli-Q water.
5. After filtering, the collected Chelex was in K^+ form and was used to purify stock electrolytes. Purification was performed by adding 80 g treated K^+ Chelex/mol dissolved K^+ salt to the electrolyte solution, and the mixture was stirred for 24 hours.
6. The mixture was then filtered, and the purified electrolyte was collected.

The following salts were used to prepare electrolytes and their stock solutions were purified: $KF \cdot 2H_2O$ (Sigma-Aldrich, 98%), KCl (Thermo-Scientific, 99.0-100.5%), and KI (Sigma-Aldrich, ≥99%).

Gases

Helium gas (Airgas, Ultra High Purity Grade) was used before experiments to purge electrolytes to remove atmospheric gases. Helium gas was also used in electrochemical mass spectrometry (EC-MS) experiments. Carbon monoxide (Airgas, Research Plus) was used as a substrate in EC-MS experiments. In addition, helium, hydrogen (Airgas, Ultra High Purity Grade), ethylene (Airgas, Research Grade), and methane (Airgas, Ultra High Purity) were used to calibrate the EC-MS. The calibration process is described in **Section 1.2**.

Cell Preparation

Before use, electrochemical cells (PTFE) were cleaned in piranha solution consisting of 75% H_2SO_4 (Sigma-Aldrich, 95.0-98.0%) and 25% H_2O_2 (Honeywell, 30% in H_2O), followed by rinsing in Milli-Q water.

Catalyst Preparation

A glassy carbon stub (Pine Research Instrumentation, 5 mm outer diameter was polished with 0.05 micron alumina suspension (Allied High Tech Products), and subsequently rinsed with Milli-Q water. After this, the stub was rinsed in concentrated H_2SO_4, followed by rinsing in concentrated HNO_3 (Sigma-Aldrich, 70% in H_2O) and then rinsing in Milli-Q water again. The glassy carbon support was then assembled into the EC-MS electrochemical cell. Copper nanopowder (Sigma-Aldrich, 99.5% trace metals basis, 25 nm) was used as the catalyst. A solution containing 0.2 mg Cu nanopowder / mL ethanol (Sigma-Aldrich, 200 proof, ≥99.5%) was placed into a vial and sonicated for 30 minutes. Following this, 10 µL of the solution was directly dropcast onto the glassy carbon and dried in an oven at 130°C for two minutes. After this, the cell was mounted onto the EC-MS system and filled with electrolyte.

1.2 Calibration of in-situ EC-MS

All experiments were performed in an EC-MS setup from SpectroInlets, Denmark. A three-electrode electrochemical cell made of PTFE was mounted onto the EC-MS and placed into contact with a membrane chip (SpectroInlets, Denmark). During electrocatalysis, gaseous products produced at the working electrode diffuse through the electrolyte to the semipermeable membrane chip. The gaseous products then diffuse through the semipermeable membrane chip and are sent to the MS where they are analyzed. The working principle of this system has been previously described elsewhere.[1] Furthermore, detailed illustrations and descriptions of the EC-MS system can be found in **Figure S1-S3**, and photos of the system are displayed in **Figure S4-S5**.

Residence Time Distribution of EC-MS

To quantify product formation using EC-MS, we began by estimating the residence time distribution (RTD) of the EC-MS system. To do so, the electrochemical cell was mounted onto the EC-MS system. A glassy carbon substrate with Cu catalyst (as described above in "Catalyst Preparation") was used as the working electrode, gold wire (Kurt J. Lesker, 99.99%) was used as the counter electrode, Ag/AgCl (BASi, 3 M KCl) was used as the reference electrode, and the electrolyte was maintained at a total K^+ concentration of 0.5 M. The RTD of the system was then determined from the decay of the of the m/z 2 signal after the applied current was set to zero. This led to the estimated

RTD of our EC-MS system, as shown in **Figure S21a**.

<u>H_2 Quantification</u>

From here, we proceeded to internally calibrate the EC-MS. Since HER can be performed with 100% Faradaic efficiency (FE), this reaction was used for the internal calibration. Using the same electrode and electrochemical system as described above to determine the RTD, we produced H_2 by performing LSV at a scan rate of 1 mV s^{-1} from −0.88 V to −1.5 V *vs* SHE in a He atmosphere in 0.5 M KF, 0.25 M KF + 0.25 M KCl, and 0.25 M KF + 0.25 M KI electrolytes. Since HER can be performed with 100% FE, we assumed that all the current from the LSV (as recorded by the potentiostat) results in H_2 formation. To obtain the internal calibration of the EC-MS, we must relate H_2 production to the m/z 2 signal. However, this is not straightforward as there is a time delay between the production of H_2 at the working electrode and the detection by the mass spectrometer. To account for this delay, we utilized the RTD of the EC-MS. Using MATLAB, we convoluted the current recorded by the potentiostat with the differential RTD of the EC-MS system. In doing so, we simulated the current in a way that accounts for the time delay of the EC-MS system. By fitting the simulated current to the background subtracted m/z 2 signal using a linear curve, we developed an internal calibration to quantify H_2 production, as exemplified in **Figure S21b**. The internal calibration was performed for each electrolyte (0.5 M KF, 0.25 M KF + 0.25 M KCl, and 0.25 M KF + 0.25 M KI) in three separate experiments. By subjecting the baseline subtracted m/z 2 ionic current from an experiment to its respective calibration curve, the data was converted into current (mA) towards H_2 production. This current is then converted into H_2 flux using the stoichiometry of the reaction and Faraday's constant. To quantify H_2 production under a CO atmosphere, the average of the three H_2 calibration values obtained under a He atmosphere for a given electrolyte was used. Unless otherwise specified, all the reported H_2 production rates from EC-MS in a CO atmosphere were quantified using the values shown in **Table S3**. Since He and CO have different dynamic viscosities, the flow rate through the chip capillary (see **Figure S1**) depends on the identity of the carrier gas.[2] To account for this, we used the Hagen-Poiseuille Equation, which describes laminar flow through a pipe:

$$\Delta P = -\frac{8\mu L}{\pi R^4} Q \tag{1}$$

where ΔP is the pressure difference across the pipe, μ is the dynamic viscosity of the carrier gas, L is the pipe length, R is the pipe radius, and Q is the flow rate.[3] To quantify H_2 production when using CO as a carrier gas, we need to determine how Q changes with respect to μ for He and CO. At the inlet of the chip capillary, the gas pressure is ~1 atm.

The outlet of the chip capillary is interfaced with a high vacuum environment (< 2E−6 mbar). Therefore, we approximate $\Delta \boldsymbol{P}$ in our system as a constant value of 1 bar. Similarly, for a given membrane chip, $\boldsymbol{L}$ and $\boldsymbol{R}$ are constant regardless of the carrier gas used. Since these values are constant, we can rearrange the Hagen-Poiseuille Equation to solve for the relative flow rate differences between CO and He atmospheres:

$$\frac{Q_{CO}}{Q_{He}} = \frac{\mu_{He}}{\mu_{CO}} \quad (2)$$

From the National Institute of Standards and Technology (NIST), at 298.15 K and 1 bar, μ_{He} = 19.846 μPa*s and μ_{CO} = 17.768 μPa*s, and so $\frac{Q_{CO}}{Q_{He}}$ = 1.1170. Therefore, to determine H_2 flux under a CO carrier gas, the baseline subtracted m/z 2 ionic current should be treated using the H_2 calibration curve obtained under He for a given electrolyte, and then multiplied by 1.1170. These internal calibrations were also necessary to quantify methane and ethylene production, as described below.

CH_4 and C_2H_4 Quantification

Products that cannot be produced with 100% FE (i.e. methane and ethylene) need to be calibrated externally by flowing known concentrations of dilute analyte gases into the EC-MS system. However, in contrast to the internal calibration described above, flowing gases past the chip of the EC-MS system (see chip design for detail) leads to a collection efficiency of less than 100%. Therefore, before externally calibrating methane and ethylene formation, it is necessary to first determine the flux of gas through the capillary of the membrane chip. This is accomplished by relating the m/z 2 signal resulting from HER to the m/z 2 signal resulting from flowing known dilute concentrations of H_2 gas into the EC-MS system, as detailed below.

Another HER internal calibration was performed, but this time using chronopotentiometry (CP). The electrochemical cell was mounted onto the EC-MS system. A Pt stub (Pine Instruments, 99.995%) was used as the working electrode, Pt wire (Kurt J. Lesker, 99.99%) was used as the counter electrode, and 1 M $HClO_4$ (Sigma Aldrich, 99.999% trace metals basis) was used as the electrolyte. A series of reductive currents were applied to the working electrode (0 μA, −2.19 μA, −2.5 μA, −3.24 μA, −3.7 μA, −4 μA, −4.22 μA, −4.3 μA, and −5.05 μA) to initiate HER, and the resulting H_2 signals (m/z 2) were recorded once they stabilized. With the assumption that HER occurs at 100% FE, the theoretical production rate of H_2 (nmol s^{-1}) at each applied current was calculated. The flux of H_2 was related to the magnitude of the background subtracted m/z 2 ionic current (A), allowing the creation of an internal H_2 calibration curve, where the slope (m) has units $\frac{nmol \cdot s^{-1}}{A}$ (**Figure S22a**). After this, six mass flow controllers (MC-Series,

Alicat Scientific) were used to dilute H_2 with He, and known concentrations of this mixture were externally flown into the EC-MS system. The resulting m/z 2 signals were compared to the signals from the internal HER calibration, allowing the creation of H_2 flux *vs* gas concentration data for the dilute regime (**Figure S22b**). By assuming that the flux of He is constant to within 0.5 mol %, calculation of the total flux of gas through the chip capillary to the MS was accomplished using the Equation below:

$$Gas\ Flux\ through\ Membrane\ Chip\ Capillary = \frac{External\ m/z\ 2\ Signal * m}{x_{H_2}} \quad (3)$$

where m is the slope from the internal calibration created using CP and $\boldsymbol{x_{H_2}}$ is the mol fraction of H_2 in He. Determining the gas flux through the capillary of the membrane chip allowed us to externally calibrate methane (m/z 15) and ethylene (m/z 26).

Using the same method as was used for diluting hydrogen, methane and ethylene gases were separately diluted with He. By flowing known concentrations of methane and ethylene into the EC-MS system and with the knowledge of the gas flux through the capillary of the membrane chip, we developed external methane and ethylene calibration curves (**Figure S22c** and **Figure S22d**).

To quantify methane and ethylene production, the H_2 calibration curves presented in **Table S3** for a given electrolyte (unless otherwise specified) were multiplied by the ratio of the external calibration slope (methane or ethylene) to the external calibration slope for hydrogen shown in **Figure S22** (unless otherwise specified). The baseline-subtracted ionic currents (m/z 15 for methane and m/z 26 for ethylene) were then subjected to these adjusted slopes. These values are then multiplied by the ratio of the number of electrons required to reduce CO to the given product by the number of electrons required to reduce water to H_2 (6/2 for methane and 8/2 for ethylene). By doing so, the ionic currents are converted to partial currents (mA) towards either methane or ethylene. These values were then converted into methane or ethylene flux using the stoichiometry of the reaction and Faraday's constant. Since the external calibrations were performed using He as the carrier gas, the resulting methane and ethylene product fluxes were multiplied by 1.1170 to account for the different dynamic viscosities of He and CO, as described earlier.

1.3 Voltammetric Experiments

A three-electrode electrochemical cell made of PTFE was used (as described above) for all voltametric experiments. The working electrode consisted of copper nanoparticles supported by glassy carbon, as described earlier. **Figure S23** shows that bare glassy carbon served solely as an inert support and was inactive for CO reduction. The counter electrode was a gold wire (Kurt J. Lesker, 99.99%) and the reference electrode was Ag/AgCl (BASi, 3 M KCl). All experiments were performed using a Biologic

SP-200 potentiostat in series with a decade box to increase the potentiostat stability. The decade box was set to a resistance of 100 Ω. The reported potentials have accounted for this resistance through post electrolysis iR correction. In addition, the reported potentials were converted from Ag/AgCl to SHE by assuming that the potential of Ag/AgCl was +0.210 V *vs* SHE. After assembling and mounting the cell onto the EC-MS, either He or CO was flowed past the chip at 1 mL/min for the duration of the experiment.

First, a constant potential of −0.88 V *vs* SHE was applied to the working electrode for 20 mins. After this, potentiostatic electrochemical impedance spectroscopy (PEIS) was performed at an applied potential of −0.88 V *vs* SHE. Following this, linear sweep voltammetry (LSV) was used to sweep the potential negatively from −0.88 V *vs* SHE to −1.5 V *vs* SHE at a scan rate of 1 mV s^{-1}.

2. Steady-state Electrolysis Experimental Procedures

2.1 Materials

Electrolyte

All electrolytes were prepared as described in **Section 1.1**.

Gases

CO (Airgas, Research Plus) was used as substrate in steady-state electrolysis experiments. Ar (Airgas, Ultra High Purity) was used to operate the gas chromatograph. The quantitative analysis was conducted using calibration curves obtained by a series of standard gas mixtures (Airgas). The standard gas uses Argon as a balanced component. The mixture included H_2 (1962 ppm), CO (1004 ppm), CH_4 (299.1 ppm), C_2H_4 (300.3 ppm) and C_2H_6 (299.1 ppm). This mixture was further diluted using Argon to obtain different concentrations. The products were quantified using an external standard curve constructed from five different concentrations.

Cell Preparation

CO reduction was conducted in a custom-designed three-chamber flow cell (the cell material was polymethyl methacrylate) manufactured by Gaossunion Co., Ltd. (structure details can be found in Ref. 4, where the CO gas was supplied directly to the catalyst layer (cathode, working electrode)).[4] All of the cell parts were cleaned using a laboratory glassware washer. First, the cell parts were washed with detergent (Miele ProCare Lab 10 AP, pH 14) at 60 to 70°C for 6 min, followed by neutralization with Miele ProCare Lab 30 C (pH 1) for 2 min and rinsed with demineralized water at 60°C. The CO gas flow rate was controlled using a mass flow controller (MC-Series, Alicat Scientific) and set to 10 sccm. Aqueous KOH solution (0.1 M) was used as the anolyte. Activated IrO_x coated Ti mesh was used as the anode (counter electrode, preparation method can be found in Ref. 5.[5] Peristaltic pumps (MasterFlex L/S, Cole-Parmer) were used to control the flow rate of the electrolytes at ~10 ml min^{-1}. A bipolar membrane (Fumasep FBM, FuMA-Tech) was used to separate the cathode and anode chambers. Electrolysis experiments were conducted using chronoamperometry with a potentiostat (Gamry Interfaces 1010E, Gamry). The cathode potentials were measured against an Ag/AgCl reference electrode (3.4 M KCl, leakless free, EDOQ ET072).

Catalyst Preparation

Freudenberg H15C13 carbon paper gas diffusion layer (GDL, Fuel Cell Store) was used as a substrate for the catalyst. The catalyst was prepared by first making a mixture consisting of 360 mg Copper nanopowder (Sigma-Aldrich, 99.5% trace metals basis, 25 nm), 40 mL isopropyl alcohol (VWR), and 108 µL Nafion perfluorinated resin solution (Sigma Aldrich, 5 wt.%). An aliquot of this mixture was then placed into an airbrush (Master Airbrush, Master Airbrush Master Performance G233 Pro Set) to deposit the catalyst onto the GDL. The aliquot of the mixture was chosen such that 2.88 mg Cu were deposited per cm^2 of carbon paper GDL (2.88 mg Cu cm^{-2} loading). After airbrushing, the Cu/GDL's were dried in an oven at 70°C for 1 hour. After this, the Cu/GDL's were ready for use.

2.2 Gas Product Analysis

The outlet of the GDE cell was connected to a gas chromatograph (SRI 8610C, SRI instruments) that is equipped with a Hayesep D and a Molecular Sieve 5Å column, thermal conductivity detector, and flame ionization detector. Ar gas was used as carrier gas. Three different potentials (−1.1, −1.3, and −1.5 V *vs* SHE) were applied to the catalyst. A new working electrode and new cathodic electrolyte were used for every measurement. Gas injections were conducted every 20 minutes for gas analysis. The

average of the two injections was reported as datapoints. The Faradaic efficiency (FE) of the gas products was calculated using the following Equation:

$$FE_{\text{gas}}\ (\%) = \frac{c_i v n F P}{R T i} \times 100\ \% \quad (4)$$

where c_i is the concentration of the gas product measured by gas chromatography (ppm), v represents the flow rate of CO ($m^3\ s^{-1}$), F is Faraday's constant (96485 C mol^{-1}), n represents the number of electrons transferred in the reaction, R is the gas constant (8.314 J mol^{-1} K^{-1}), T is temperature (K), P is the pressure in the electrochemical cell headspace (Pa), and i is the current (A).

2.3 Liquid Product Analysis

To analyze the liquid products, the post-electrolysis solutions were obtained through the same method as described above. 400 μL of electrolyte was mixed with 100 μL of a solution of 10 mM dimethyl sulfoxide (DMSO) and 50 mM phenol in D_2O as internal standards for ^{1}H-NMR analysis. The internal standards, phenol and DMSO, were chosen because they did not interfere with peaks arising from CO reduction products and because of their non-volatility which allowed for use and storage of the same internal standards solution for all of the product measurements without appreciable change in concentration. The area of product peaks to the right of the water peak was compared to the area of DMSO (at a chemical shift of 2.6 ppm), and the area of product peaks to the left of the water peak was compared to the area of phenol (at a chemical shift of 7.2 ppm).[6,7] To avoid the loss of highly volatile species, all electrolytes containing liquid products were stored in a refrigerator (4℃). ^{1}H NMR spectra of the post-electrolysis solution were recorded using a 400 MHz NMR (Bruker Avance) with a BBFO probe. Water suppression was utilized to reduce the intensity of the water peak (4.79 ppm). The integrated area for the liquid products, n-propanol (0.77 ppm, triplet), ethanol (1.06 ppm, triplet), and acetate (1.87 ppm, singlet) were calculated to get the moles of the liquid product (N_i). The Faradic efficiency of the liquid products was calculated using the following Equation:

$$FE_{\text{liq}}\ (\%) = \frac{N_i n F}{Q_{\text{total}}} \times 100\ \% \quad (5)$$

where Q_{total} is the total charge passed during the electrolysis.

3. Calculation of Local pH

The local concentration of OH^- in the electrolyte was simulated using a 1D reaction diffusion model.[7,8] Film theory is assumed to be applicable where, in the concentration boundary layer, the velocity gradients or convective effects are assumed to be negligible.[8] The following balances would then occur within a slice of solution from *x* to *x+Δx* in **Figure S24**. The governing Equations:

$$\frac{\partial[OH]}{\partial t} = D_{OH}\frac{\partial^2[OH]}{\partial x^2} - R_{OH} \tag{6}$$

D_{OH} is the is the diffusion coefficient of OH^- (5.273×10^{-9} m^2 s^{-1}).[8] The OH^- formation on the electrode surface can be estimated as:

$$R_{OH} = \frac{j}{F} \tag{7}$$

j is the geometric current density of Cu nanoparticles in A/m^2, *F* is Faraday's constant (96485 C mol^{-1}).

The above Equations are second-order time-dependent partial differential equations that are to be solved under the following boundary conditions. The initial values of the concentrations (at $t = 0$, before current flows) are assumed to be the same as in the bulk solution and are listed in **Table S4** for different electrolytes.

At time $t > 0$ and $x = 0$ (i.e., at the interface of bulk solution and the boundary layer):

$$[OH] = [OH]_B \tag{8}$$

where $[OH]_B$ are the equilibrium values in the bulk solution, which are the same as the values given in **Table S4**.

At time $t > 0$ and $x = \delta$ (x-direction coordinates at the position of interest, e.g. electrode surface) are related to the reaction fluxes (i.e., the electrode surface is an impermeable, reflective wall for all species):

$$\frac{\partial[OH]}{\partial x} = R_{OH} \tag{9}$$

With all the boundary conditions and constants known, the partial differential equations were solved using MATLAB software.

4. Supplemental Figures and Tables

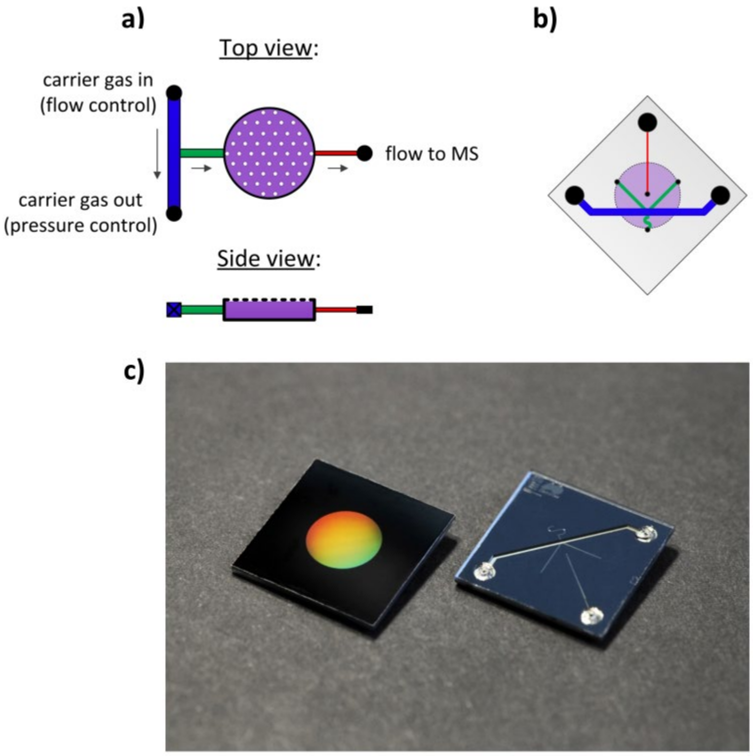

Figure S1: Membrane chip design used in the EC-MS setup. **(a)** Illustration describing how the membrane chip works. The carrier gas (i.e. He or CO) channels are shown in blue, the delivery channel for the carrier gas to the sampling volume is shown in green, the sampling volume is shown in purple, and the capillary is shown in red. The black circles indicate the interfaces between the membrane chip and the external vacuum and gas systems. **(b)** Illustration describing the design of the gas system of the membrane chip. **(c)** Image of the membrane chip. The image on the left displays the membrane of the membrane chip, and the image on the right displays the gas channels of the membrane chip. Reprinted from Electrochimica Acta, 268, Trimarco, D.B., Scott, S.B., Thilsted, A.H., Pan, J.Y., Pedersen, T., Hansen, O., Chorkendorff, I., Vesborg, P.C.K., Enabling real-time detection of electrochemical desorption phenomena with sub-monolayer sensitivity, 520–530, Copyright (2018), with permission from Elsevier.

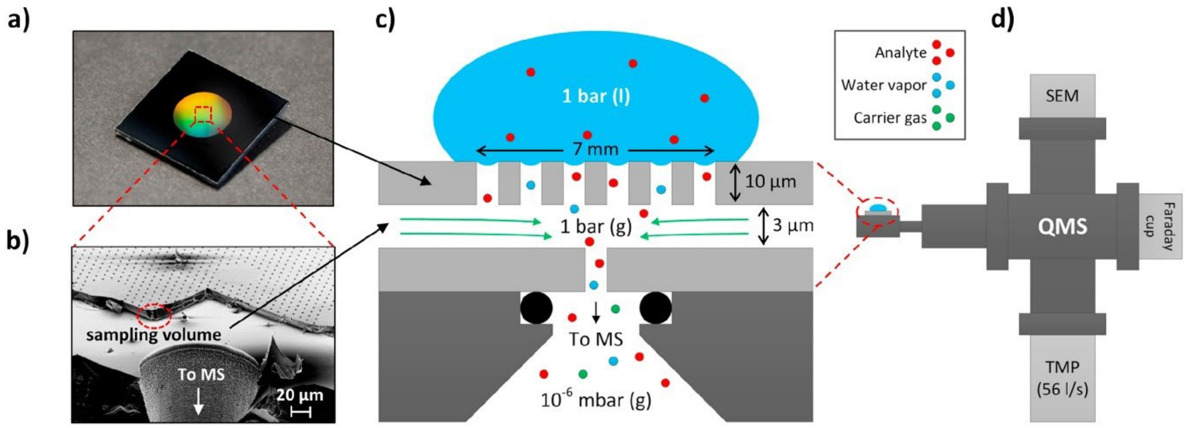

Figure S2: Membrane chip and working principle of the EC-MS. **(a)** Image of the membrane chip that is required for the EC MS setup. **(b)** Scanning electron microscope image of the structure of the membrane chip. The red circle shows the sampling volume of the membrane chip. **(c)** Illustration of a water droplet (blue) on top of the membrane chip. The water droplet contains dissolved analyte (red). Carrier gas (i.e. He or CO, green) flows through the sampling volume of the membrane chip, and then flows into both the water droplet as well as into the vacuum chamber. Water vapor, analyte, and the carrier gas are sent from the sampling volume of the membrane chip through its capillary to the vacuum chamber where the species are analyzed by the mass spectrometer (MS). **(d)** Illustration of the membrane chip (grey) mounted on the MS system. Reprinted from Electrochimica Acta, 268, Trimarco, D.B., Scott, S.B., Thilsted, A.H., Pan, J.Y., Pedersen, T., Hansen, O., Chorkendorff, I., Vesborg, P.C.K., Enabling real-time detection of electrochemical desorption phenomena with sub-monolayer sensitivity, 520–530, Copyright (2018), with permission from Elsevier.

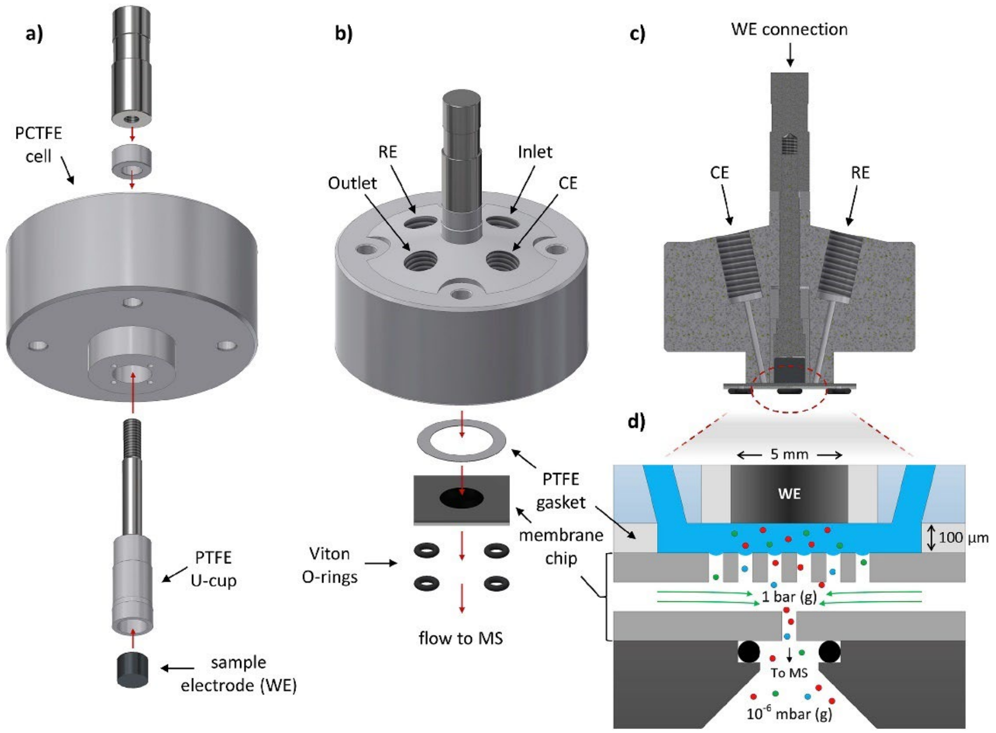

Figure S3: Schematic illustration of the electrochemical cell used in EC-MS. **(a)** Assembly of the electrochemical cell. Cell assembly requires inserting a 5.0 mm diameter glassy carbon working electrode into a rotating disc electrode (RDE) mounting system from Pine Research. Note that a PTFE cell was used in our study instead of a PCTFE cell. **(b)** Mounting of the electrochemical cell onto the EC-MS system. A 100 μm thick PTFE gasket is used to define a thin layer of electrolyte between the electrode and the membrane chip (working volume electrolyte). The membrane chip is sealed to the ultra high vacuum system of the MS using Viton O rings. The working volume electrolyte is connected to the rest of the electrochemical cell via four access channels. The access channels hold the counter and reference electrodes, as shown in **Figure S4**. **(c)** and **(d)** show a side cut view of how the working electrode, **(c)**, contacts the working volume electrolyte (100 μm thick) and the membrane chip, **(d)**. Reprinted from Electrochimica Acta, 268, Trimarco, D.B., Scott, S.B., Thilsted, A.H., Pan, J.Y., Pedersen, T., Hansen, O., Chorkendorff, I., Vesborg, P.C.K., Enabling real-time detection of electrochemical desorption phenomena with sub-monolayer sensitivity, 520–530, Copyright (2018), with permission from Elsevier.

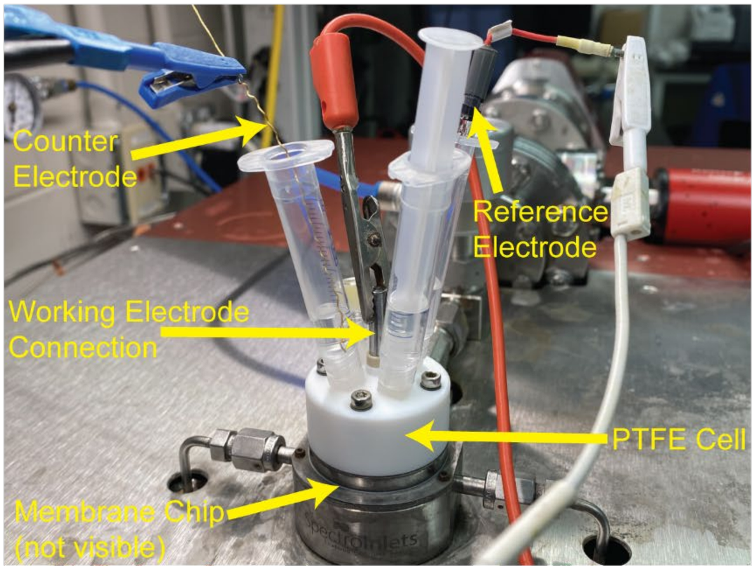

Figure S4: Image of the three-electrode electrochemical cell (PTFE) mounted on the EC-MS.

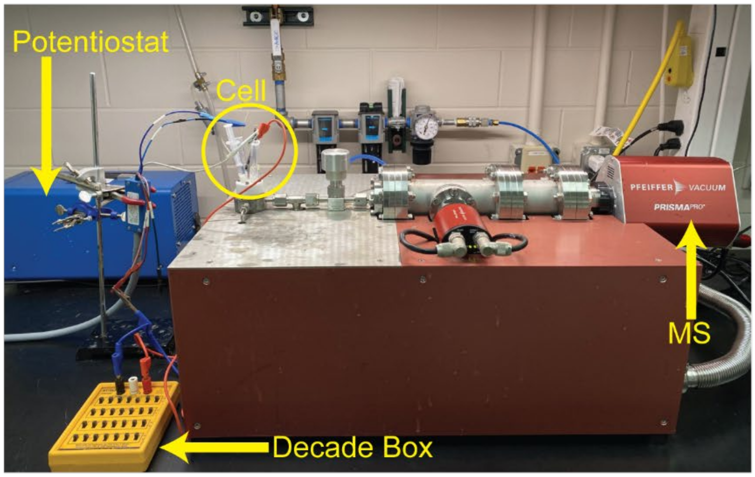

Figure S5: Image of the EC-MS setup which includes a potentiostat, a decade box, a three-electrode electrochemical cell, and a mass spectrometer (MS).

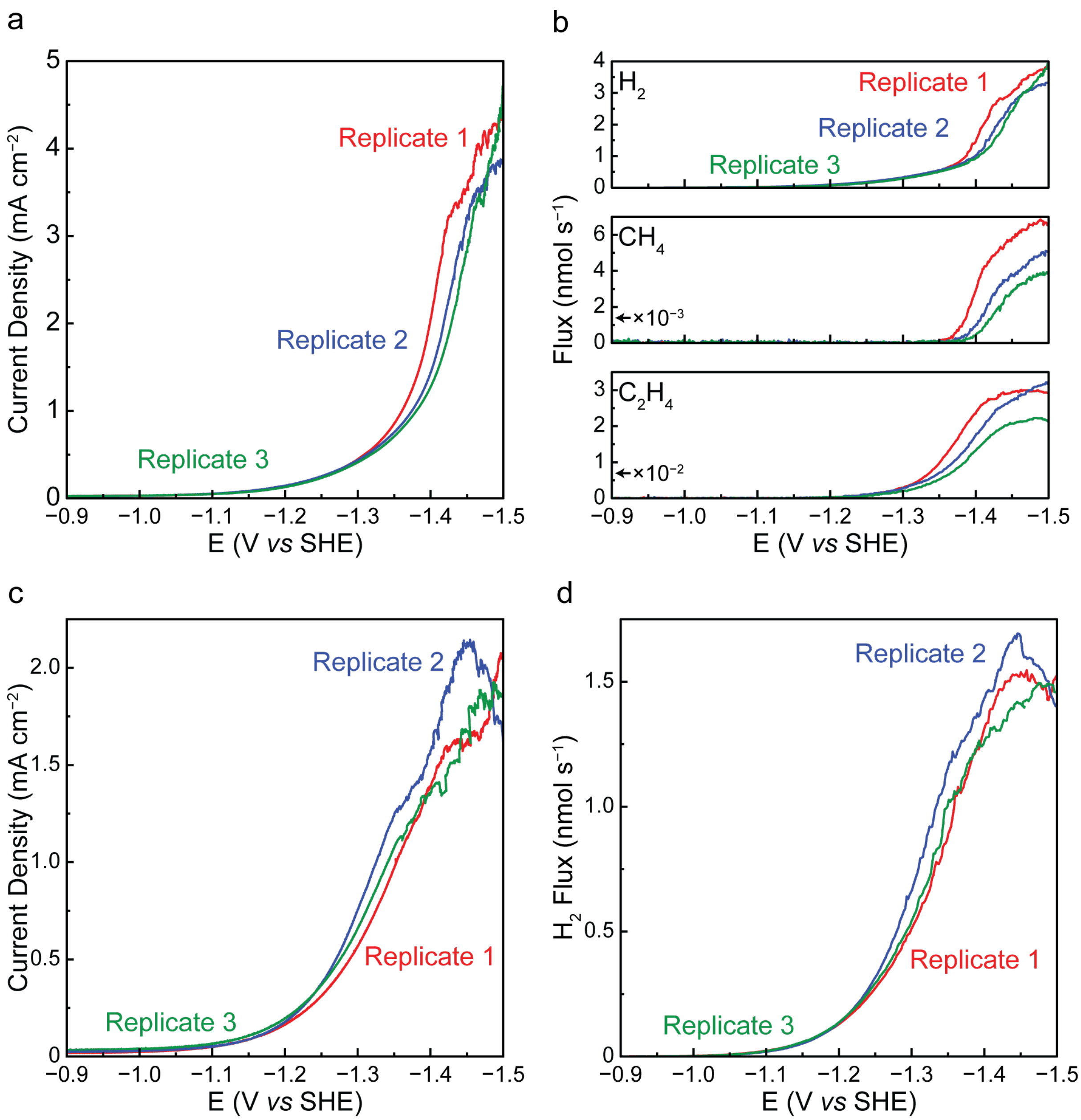

Figure S6: Replicates of LSV experiments performed at a scan rate of 1 mV s^{-1} in 0.5 M KF. Each experiment was replicated three separate times using fresh electrolyte and an independent Cu catalyst (prepared as described in **1.1 Materials**). The colors of the curves represent different replicates. **(a)** Current densities during LSV in the presence of CO and **(b)** the corresponding fluxes of H_2, CH_4, and C_2H_4. **(c)** Current densities during LSV in the presence of He and **(d)** the corresponding flux of H_2. The averaged FE of the replicated trials under CO and He are shown in **Figure S25** and **Figure S26**, respectively.

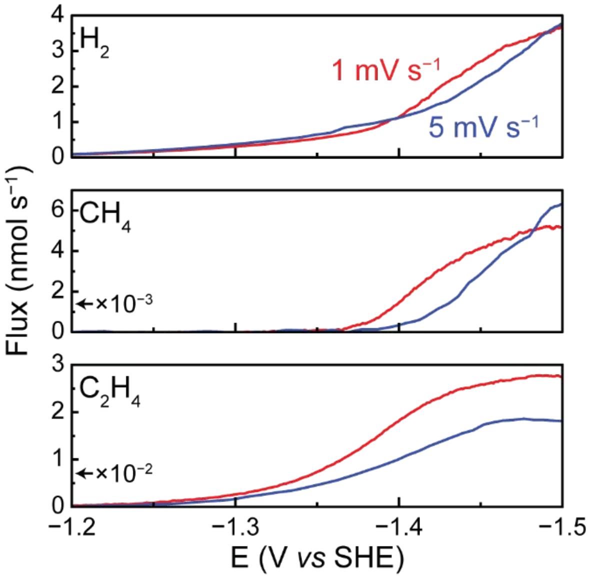

Figure S7: LSV experiments performed at scan rates of 1 mV s^{-1} and 5 mV s^{-1} in 0.5 M KF under a CO atmosphere. The 1 mV s^{-1} data is an average of three independent experiments (replicates shown in **Figure S6**) and the 5 mV s^{-1} data is an average of two independent experiments.

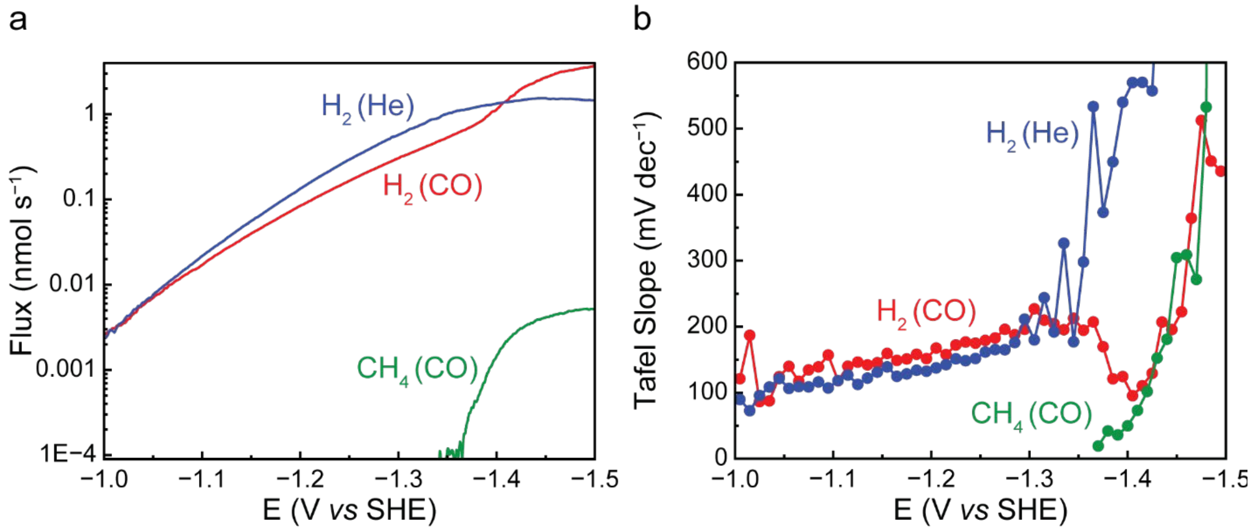

Figure S8: Correspondence between the onset of CH_4 production and the change in the HER mechanism. LSV experiments were performed at a scan rate of 1 mV s^{-1} in 0.5 M KF in the presence of CO or He. The presented data is an average of three independent experiments (replicates shown in **Figure S6**). **(a)** Production rates of hydrogen and methane in the presence of CO or He. **(b)** Tafel slope plot for H_2 production in the presence of CO and He atmospheres, as well as the Tafel slope plot for CH_4 production in the presence of CO.[9] The potential range used to create the Tafel slope plot was 10 mV.[9]

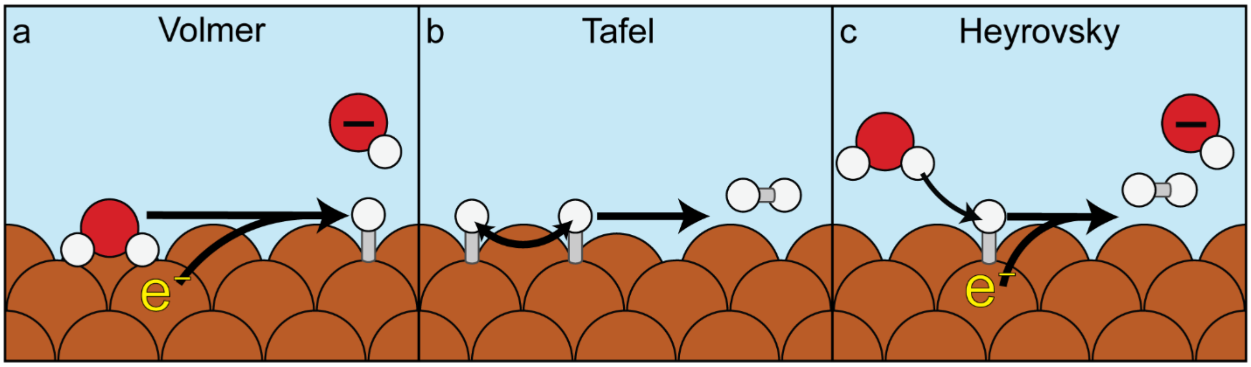

Figure S9: Elementary steps in HER. H_2 production can follow either a Volmer-Tafel pathway (**a** followed by **b**) or a Volmer-Heyrovsky pathway (**a** followed by **c**). **(a)** The Volmer step involves electron transfer to dissociate water and form H* and OH^-. **(b)** The Tafel step is the chemical coupling between 2 H* to form H_2. **(c)** The Heyrovsky step involves proton and electron between water and H* to form H_2 and OH^-.

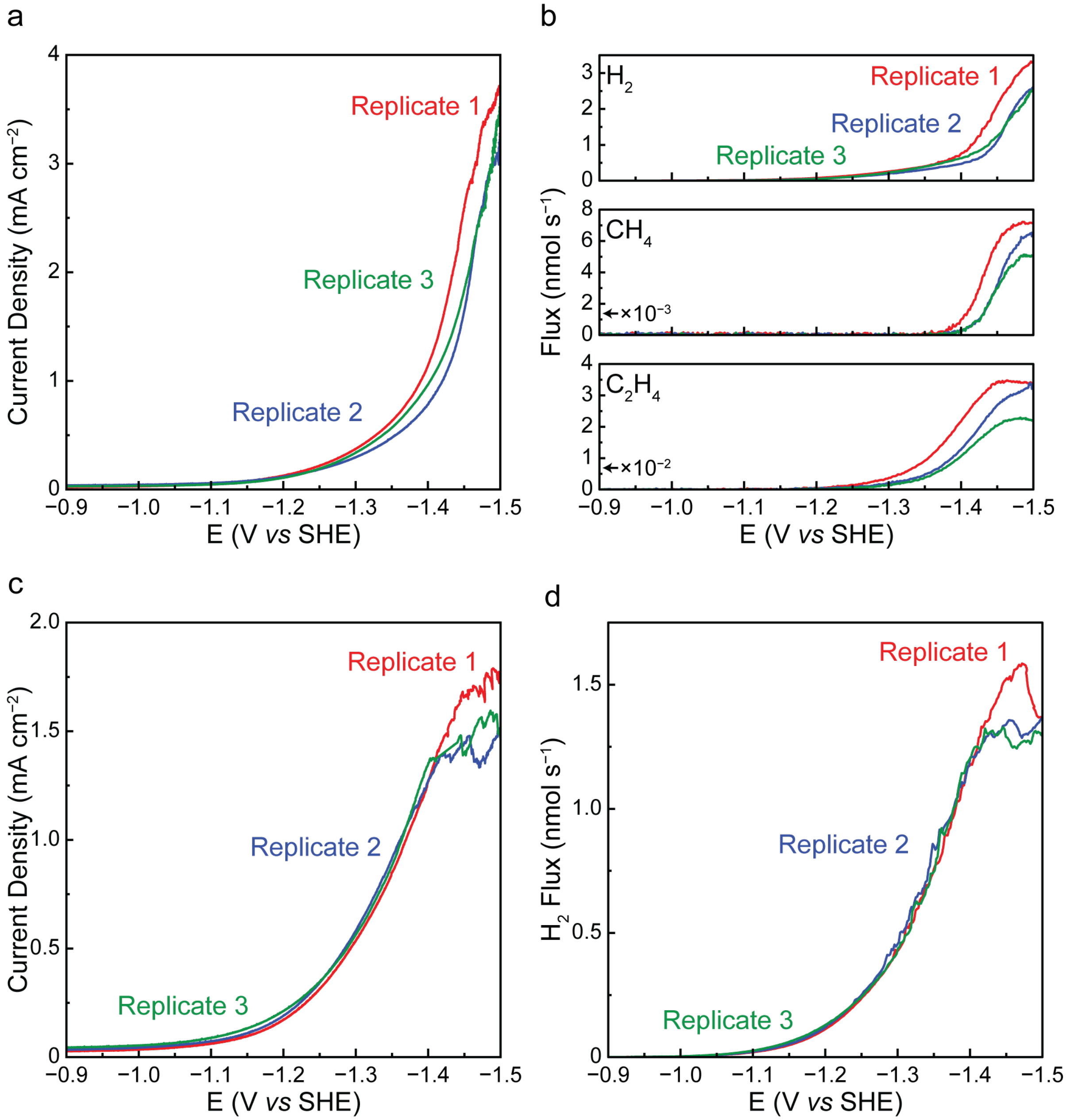

Figure S10: Replicates of LSV experiments performed at a scan rate of 1 mV s^{-1} in 0.25 M KF + 0.25 M KCl. Each experiment was replicated three separate times using fresh electrolyte and an independent Cu catalyst (prepared as described in **1.1 Materials**). The colors of the curves represent different replicates. **(a)** Current densities during LSV in the presence of CO and **(b)** the corresponding fluxes of H_2, CH_4, and C_2H_4. **(c)** Current densities during LSV in the presence of He and **(d)** the corresponding flux of H_2. The averaged FE of the replicated trials under CO and He are shown in **Figure S25** and **Figure S26**, respectively.

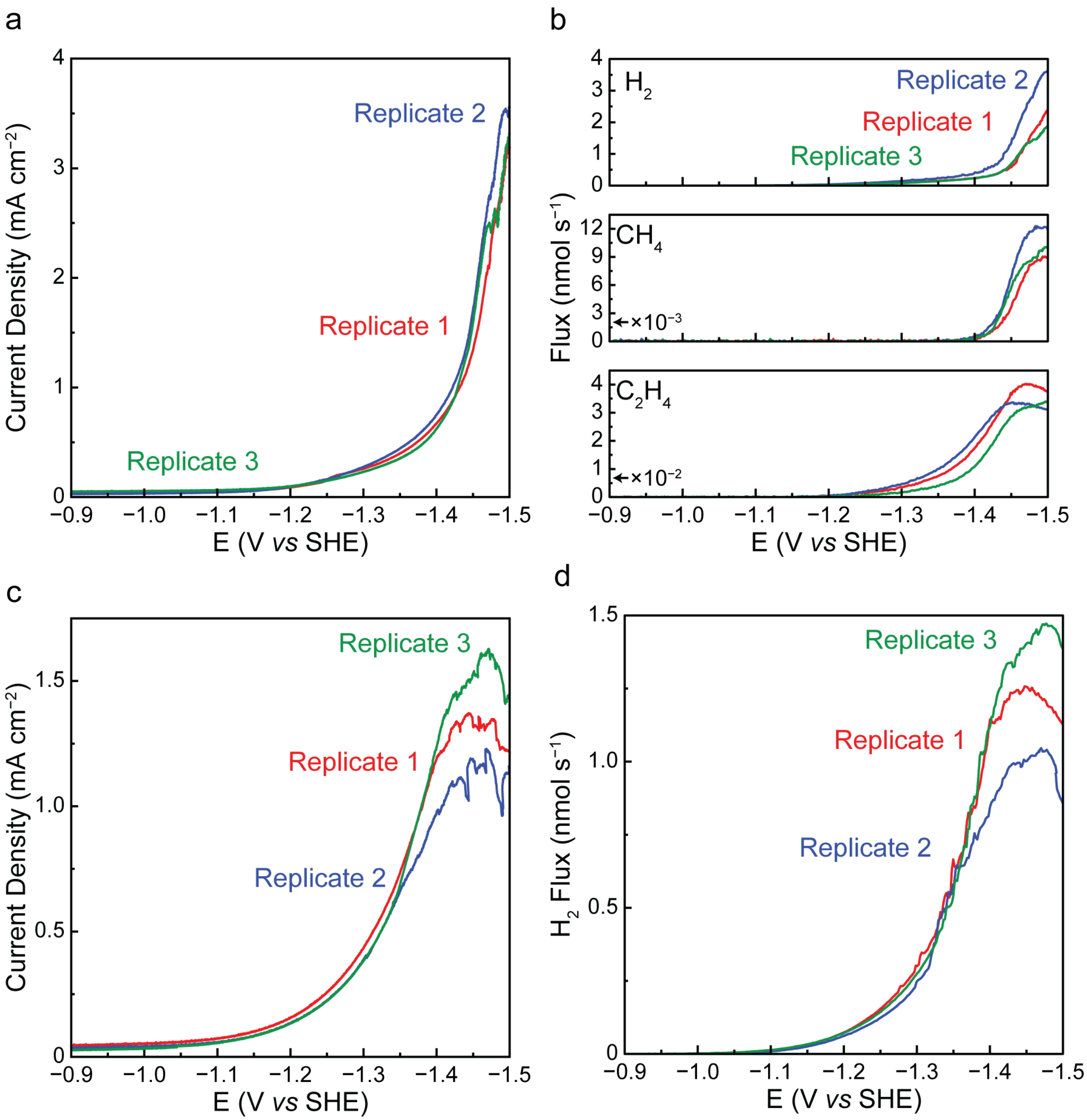

Figure S11: Replicates of LSV experiments performed at a scan rate of 1 mV s^{-1} in 0.25 M KF + 0.25 M KI. Each experiment was replicated three separate times using fresh electrolyte and an independent Cu catalyst (prepared as described in **1.1 Materials**). The colors of the curves represent different replicates. **(a)** Current densities during LSV in the presence of CO and **(b)** the corresponding fluxes of H_2, CH_4, and C_2H_4. **(c)** Current densities during LSV in the presence of He and **(d)** the corresponding flux of H_2. The averaged FE of the replicated trials under CO and He are shown in **Figure S25** and **Figure S26**, respectively.

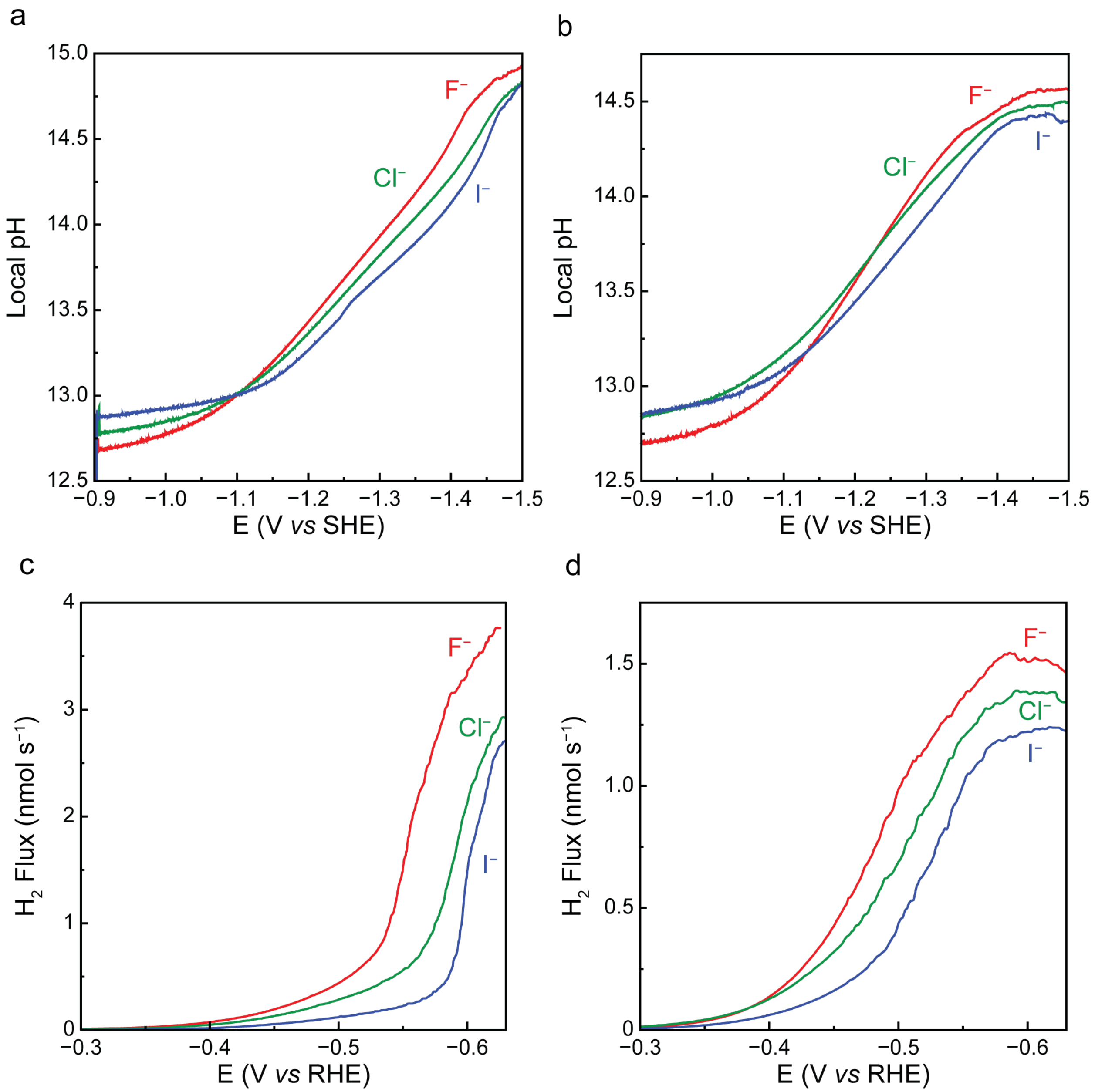

Figure S12: A reaction diffusion model was used to calculate the evolution of the local pH as a function of the applied potential. LSV was performed at a scan rate of 1 mV s^{-1} under CO or He in the following electrolytes: 0.5 M KF, 0.25 M KF + 0.25 M KCl, and 0.25 M KF + 0.25 M KI. The local pH calculations were performed using the average of three independent experiments for each electrolyte (replicates shown in **Figure S6**, **Figure S10**, and **Figure S11**). **(a)** Local pH changes during LSV in a CO atmosphere. **(b)** Local pH changes during LSV in a He atmosphere. **(c)** The calculated local pH in a CO atmosphere was used to plot the H_2 flux in a CO atmosphere against the local pH corrected reversible hydrogen electrode (RHE). **(d)** The calculated local pH in a He atmosphere was used to plot the H_2 flux in a He atmosphere against the local pH corrected RHE.

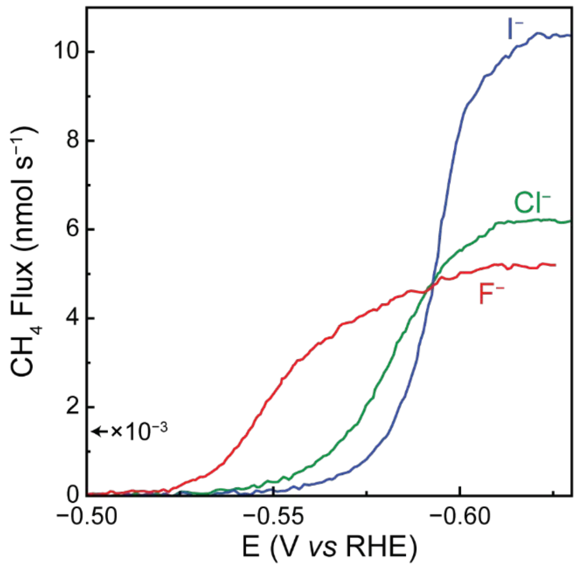

Figure S13: The calculated local pH in a CO atmosphere was used to plot CH_4 flux against the local pH corrected RHE in different electrolytes. A reaction diffusion model was used to calculate the evolution of the local pH as a function of the applied potential. LSV was performed at a scan rate of 1 mV s^{-1} under CO in the following electrolytes: 0.5 M KF, 0.25 M KF + 0.25 M KCl, and 0.25 M KF + 0.25 M KI. The local pH calculations were performed using the average of three independent experiments for each electrolyte (replicates shown in **Figure S6**, **Figure S10**, and **Figure S11**).

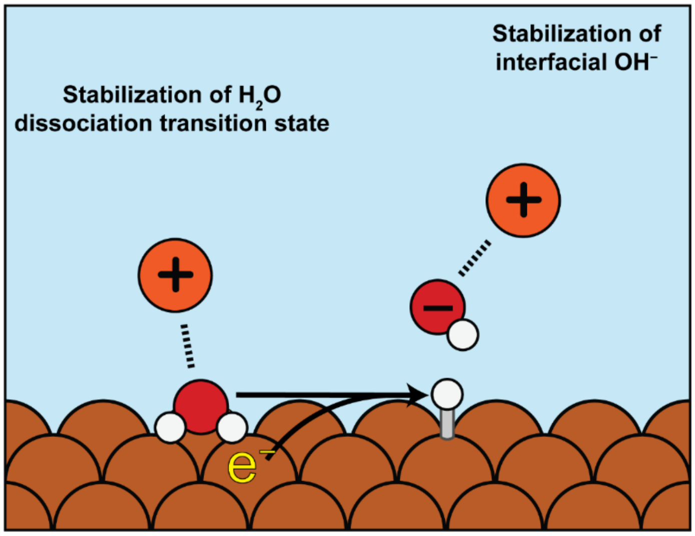

Figure S14: In neutral and alkaline electrolytes on Cu, it has been suggested that cations promote hydrogen production by stabilizing the transition state of water dissociation to form H*, as well as by stabilizing the formation of interfacial OH^-.[10]

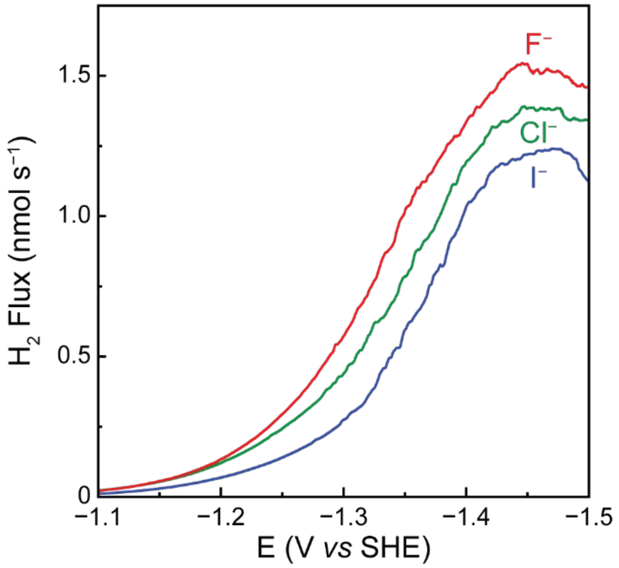

Figure S15: EC-MS analysis shows the production rates of H_2. LSV was performed at a scan rate of 1 mV s^{-1} under He in the following electrolytes: 0.5 M KF, 0.25 M KF + 0.25 M KI and 0.25 M KF + 0.25 M KCl. The presented data is an average of three independent experiments (replicates shown in **Figure S6**, **Figure S10**, and **Figure S11**).

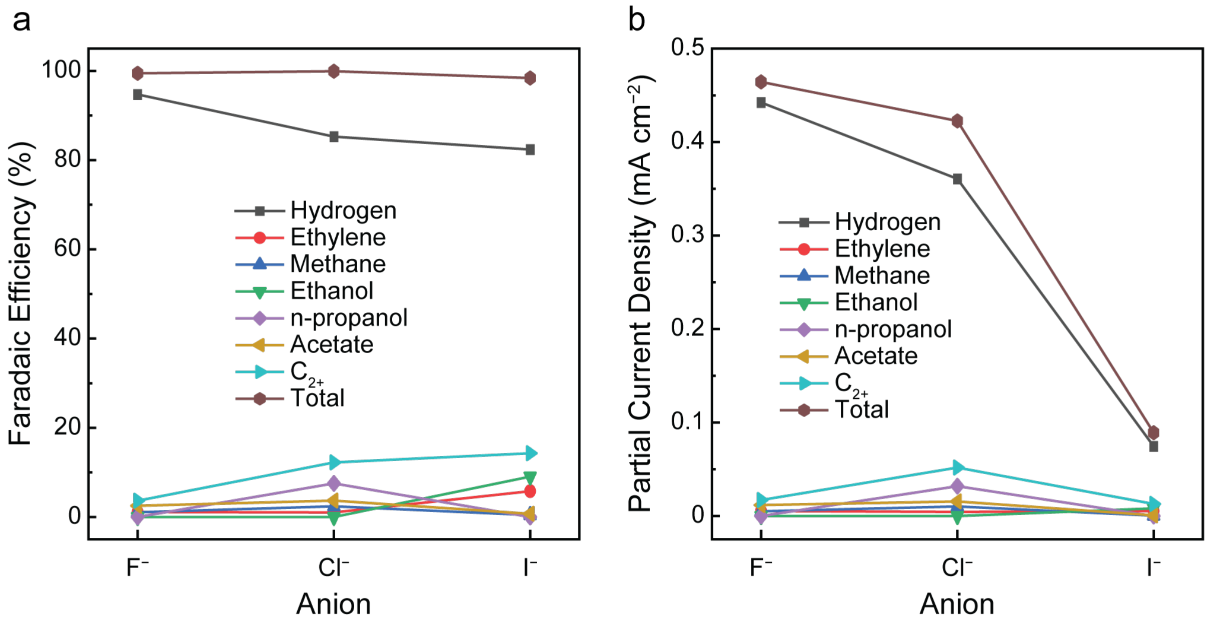

Figure S16: Chronoamperometry was performed at −1.1 V in a flow cell using a gas diffusion electrode loaded with Cu catalyst. Experiments were performed in the presence of CO in 0.5 M KF, 0.25 M KF + 0.25 M KCl, and 0.25 M KF + 0.25 M KI. The resulting CO reduction products were quantified by GC and ^{1}HNMR. **(a)** The FEs and **(b)** partial current densities towards various products are shown.

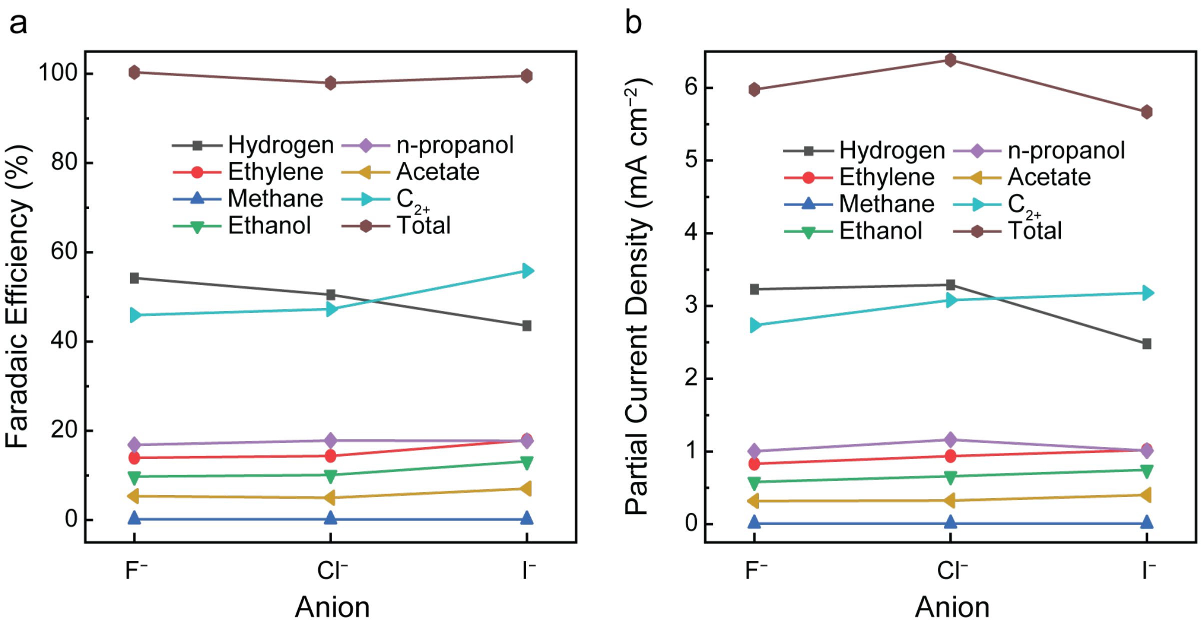

Figure S17: Chronoamperometry was performed at −1.3 V in a flow cell using a gas diffusion electrode loaded with Cu catalyst. Experiments were performed in the presence of CO in 0.5 M KF, 0.25 M KF + 0.25 M KCl, and 0.25 M KF + 0.25 M KI. The resulting CO reduction products were quantified by GC and ^{1}HNMR. **(a)** The FEs and **(b)** partial current densities towards various products are shown.

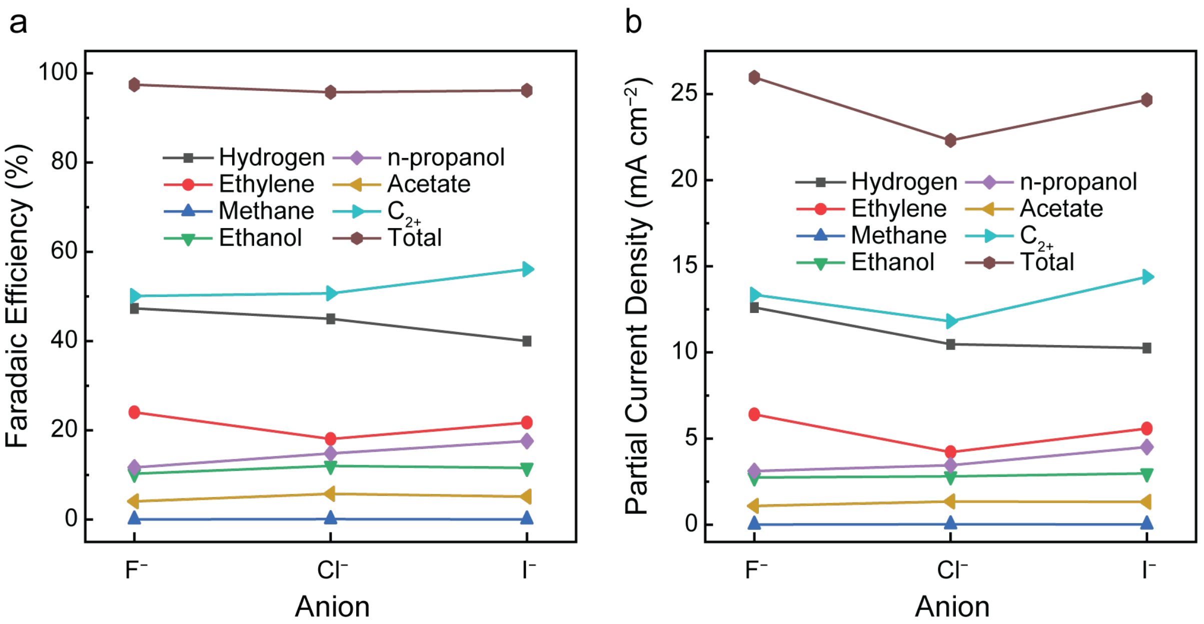

Figure S18: Chronoamperometry was performed at −1.5 V in a flow cell using a gas diffusion electrode loaded with Cu catalyst. Experiments were performed in the presence of CO in 0.5 M KF, 0.25 M KF + 0.25 M KCl, and 0.25 M KF + 0.25 M KI. The resulting CO reduction products were quantified by GC and ^{1}HNMR. **(a)** The FEs and **(b)** partial current densities towards various products are shown.

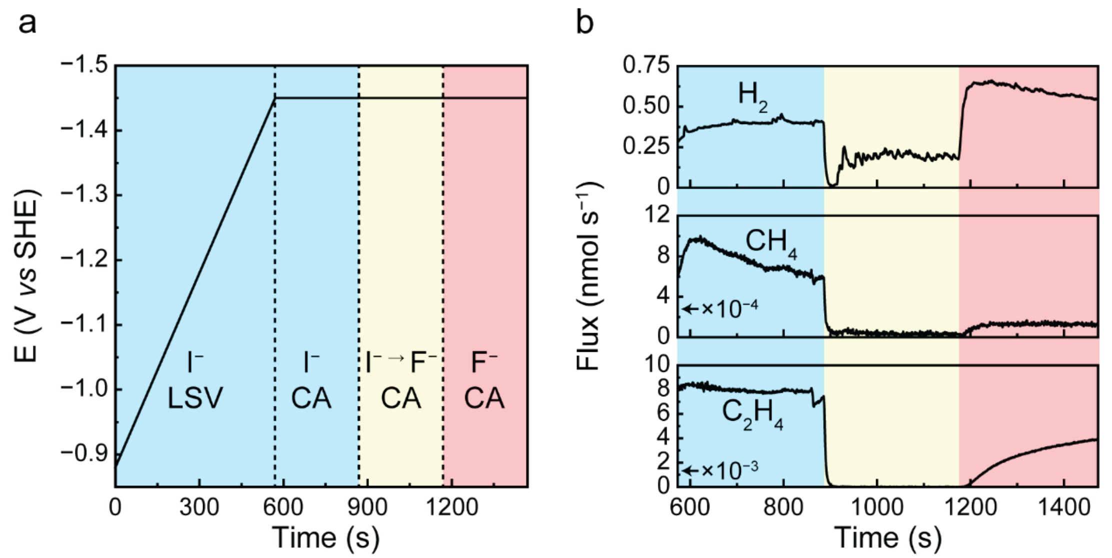

Figure S19: Electrolyte exchange experiment. This experiment was performed to see if halide-induced Cu reconstruction could influence the experimental results. **(a)** Overview of the experimental protocol. This experiment began using 0.25 M KF + 0.25 M KI electrolyte and CO as a substrate. Initially, −0.88 V (all potentials reported vs SHE) was applied to the working electrode for 20 minutes, followed by PEIS at −0.88 V (these steps are not shown schematically above). After this, LSV was performed (starting at time = 0 s in the figure above) at a scan rate of 1 mV s^{-1} from −0.88 V to −1.45 V. Once the potential reached −1.45 V, it was held constant for the rest of the experiment. For the first 300 seconds of applying −1.45 V, the electrolyte was left untouched. After this, while continuously applying −1.45 V, 0.25 M KF + 0.25 M KI electrolyte is removed from the EC-MS cell and replaced with 0.5 M KF (yellow shaded region). This is done by using a syringe pump to flow 5 mL of 0.5 M KF into the EC-MS cell at a rate of 1 mL min^{-1}. After 300 seconds, the 0.25 M KF + 0.25 M KI electrolyte is fully replaced by 0.5 M KF (red shaded region). **(b)** The production rates of hydrogen, methane, and ethylene during CA at −1.45 V. The colors in this plot match the colors and corresponding description in **(a)**. Blue corresponds to CA at −1.45 V in the presence of 0.25 M KF + 0.25 M KI. Yellow corresponds to CA at −1.45 V while performing electrolyte exchange from 0.25 M KF + 0.25 M KI to 0.5 M KF. Red corresponds to CA at −1.45 V in the presence of 0.5 M KF. Note that in the yellow shaded region, the production rates of H_2, CH_4, and C_2H_4 drop significantly, and then increase again in the red shaded region. The reason for the decreased production rates in the yellow shaded region is likely due to the electrolyte being flown out of the cell, resulting in a portion of the H_2, CH_4, and C_2H_4 produced also being flowed out of the cell rather than diffusing through the membrane chip and entering the mass spectrometer. The calibration used for this experiment was similar to what is described earlier, but the simulated current was fitted to the background subtracted m/z 2 signal using a second order polynomial, which was used to quantify H_2 production. Furthermore, instead of using the internal HER calibration obtained from the second order polynomial fitting, CH_4 (m/z 15) and C_2H_4 (m/z 26) were subjected to an internal H_2

calibration that was performed in the linear response region of the EC-MS instrument.

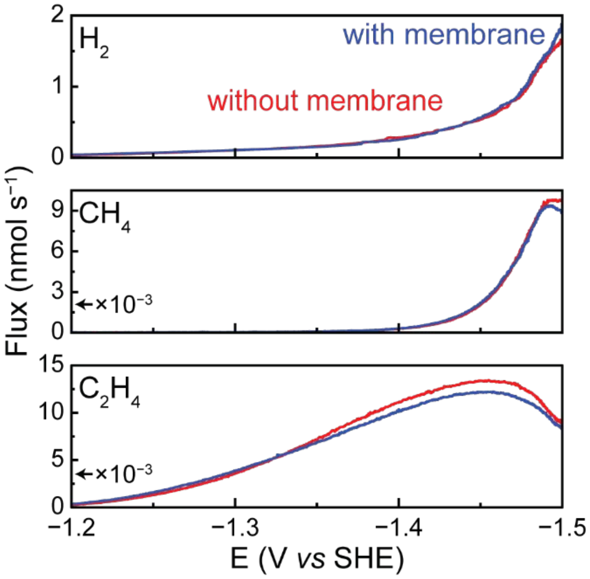

Figure S20: LSV was performed at a scan rate of 1 mV s^{-1} under CO with and without a proton exchange membrane (PEM, PFSA D170-U, Fuel Cell Store) in 0.25 M KF + 0.25 M KI. Each experiment (with and without membrane) was replicated two times using fresh electrolyte and an independent Cu catalyst (prepared as described in **1.1 Materials**). The presented data is an average of the two replicates. The calibration used for this experiment was similar to what is described earlier, but the simulated current was fitted to the background subtracted m/z 2 signal using a second order polynomial, which was used to quantify H_2 production. Furthermore, instead of using the internal HER calibration obtained from the second order polynomial fitting, CH_4 (m/z 15) and C_2H_4 (m/z 26) were subjected to an internal H_2 calibration that was performed in the linear response region of the EC-MS instrument.

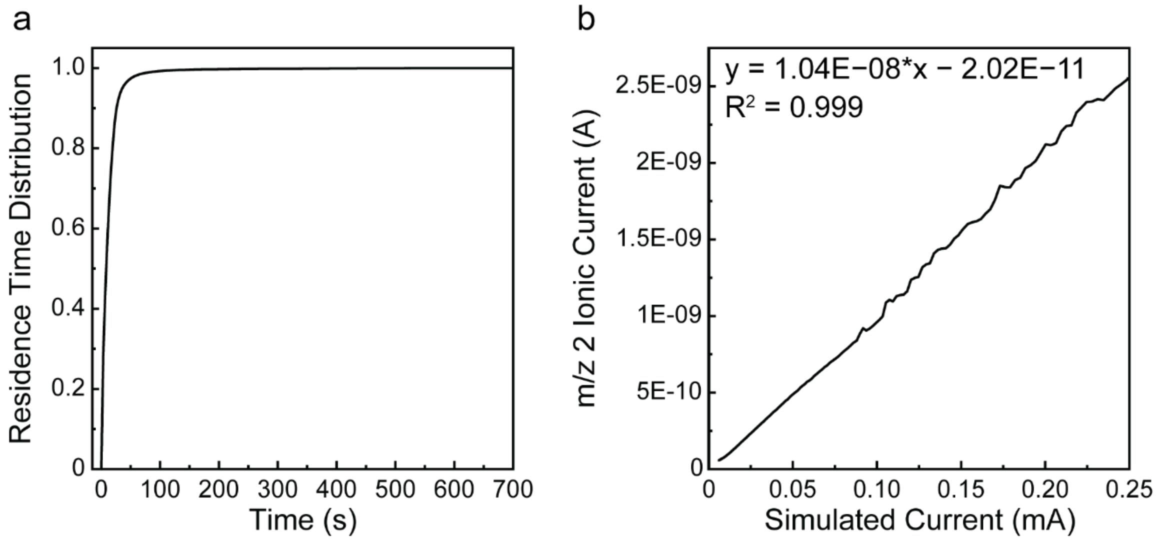

Figure S21: (a) Measured residence time distribution (RTD) of the EC-MS system. **(b)** A representative example of a H_2 (m/z 2) internal calibration for the EC-MS system. See **1.2 Calibration of in-situ EC-MS** for details on determination of both the RTD and the internal calibration.

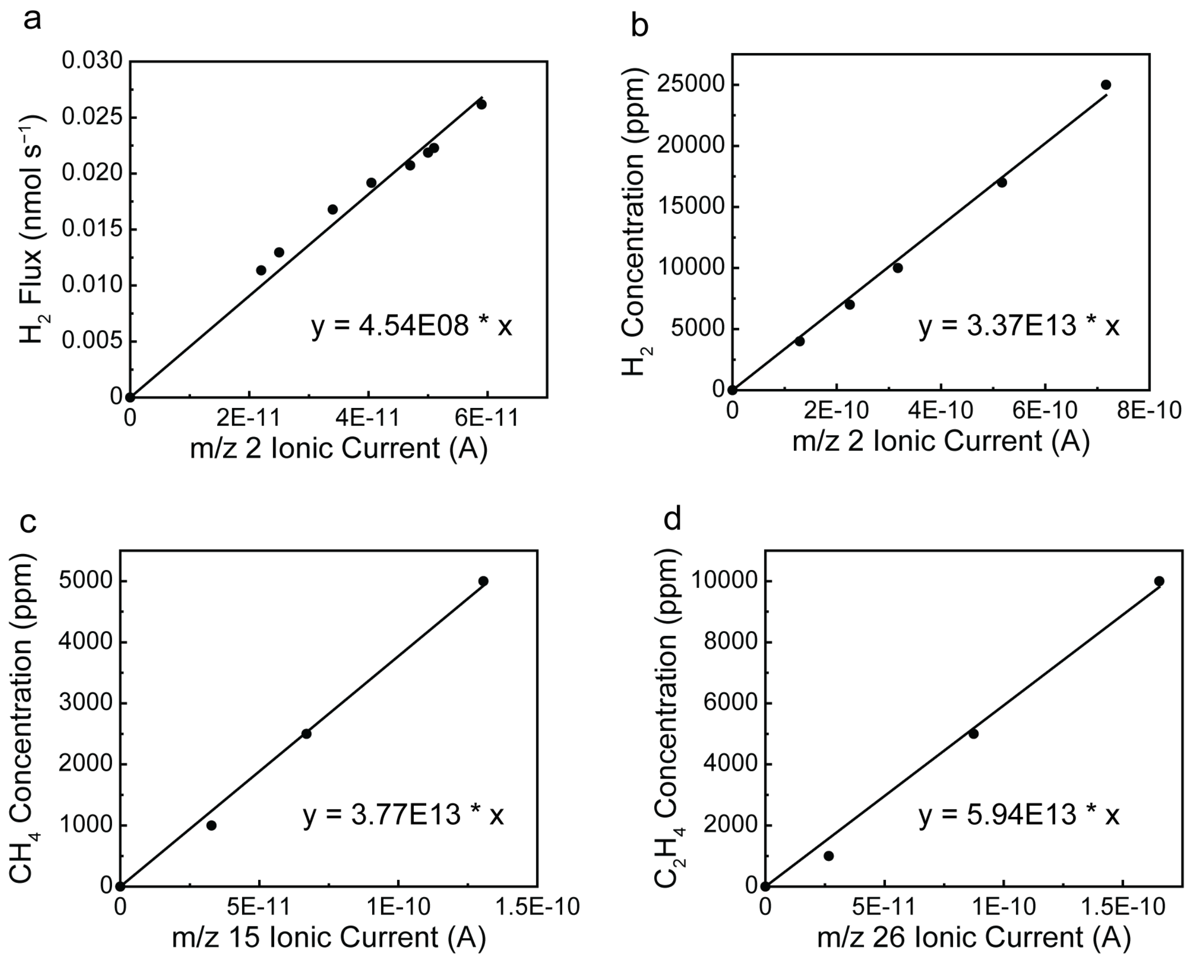

Figure S22: Calibration curves used to quantify the amount of CH_4 and C_2H_4 produced in this study. **(a)** H_2 internal calibration using chronopotentiometry to determine the flux of gas through membrane chip, **(b)** H_2 external calibration, **(c)** CH_4 external calibration, and **(d)** C_2H_4 external calibration. See **1.2 Calibration of in-situ EC-MS** for details on the internal calibration.

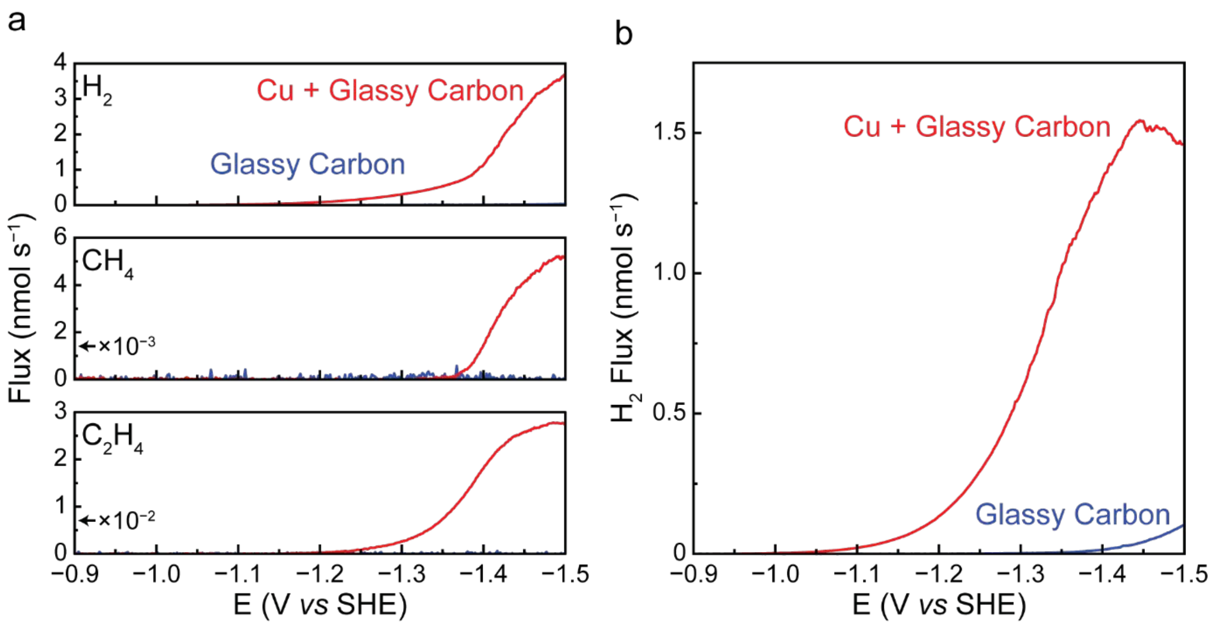

Figure S23: LSV performed at a scan rate of 1 mV s^{-1} in 0.5 M KF with and without Cu nanoparticles dropcast onto glassy carbon, demonstrating that glassy carbon is inactive for CO reduction. **(a)** Production of H_2, CH_4, and C_2H_4 under CO atmosphere. **(b)** Production of H_2 under He atmosphere.

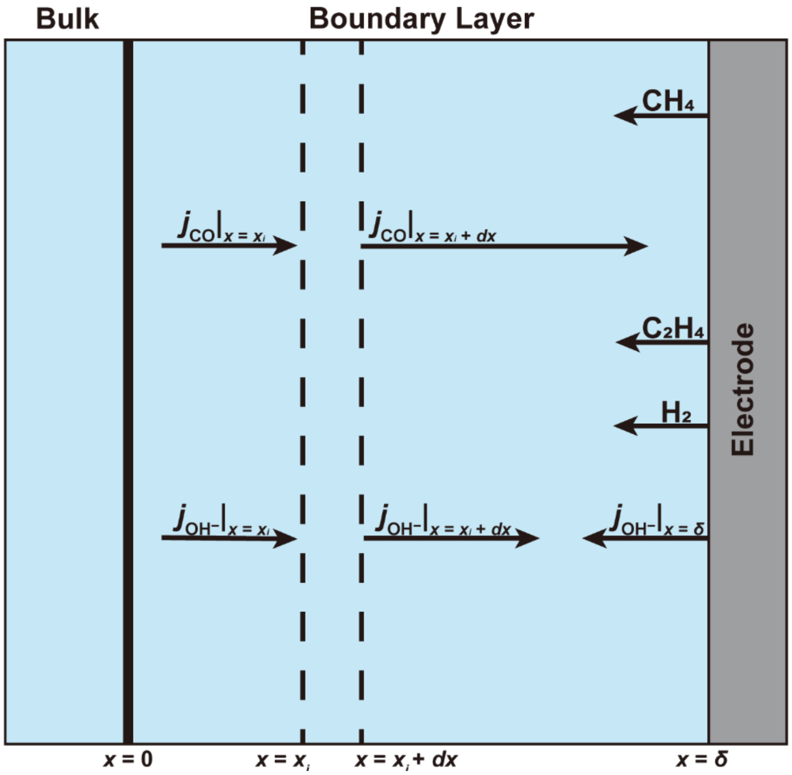

Figure S24: Illustration of the mass transport model for the electroreduction of CO on Cu. The symbol δ in the figure represents the thickness of the boundary layer, which is set to 100 μm in this work.

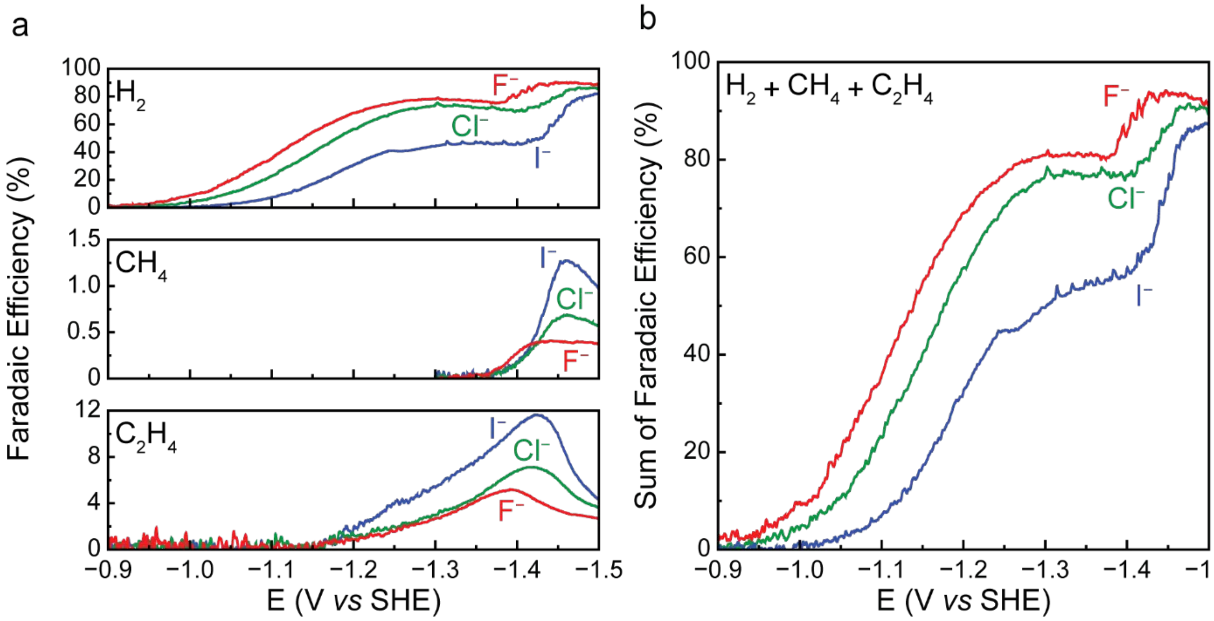

Figure S25: Faradaic efficiency during LSV under CO. LSV was performed at a scan rate of 1 mV s^{-1} under CO in the following electrolytes: 0.5 M KF, 0.25 M KF + 0.25 M KCl, and 0.25 M KF + 0.25 M KI. The FE's were calculated using the average of three independent experiments for each electrolyte (replicates shown in **Figure S6**, **Figure S10**, and **Figure S11**). **(a)** The FEs towards H_2, CH_4, and C_2H_4 are recorded as a function of potential. **(b)** The sum of the FEs towards H_2, CH_4, and C_2H_4 are recorded as a function of potential.

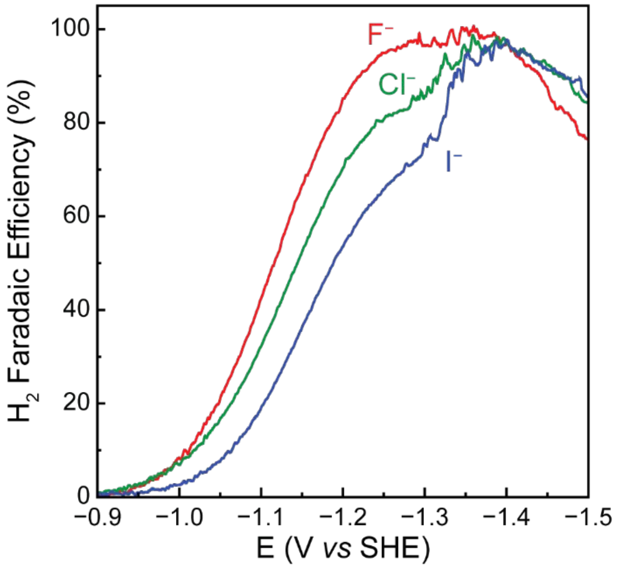

Figure S26: Faradaic efficiency during LSV under He. LSV was performed at a scan rate of 1 mV s^{-1} under He in the following electrolytes: 0.5 M KF, 0.25 M KF + 0.25 MKCl, and 0.25 M KF + 0.25 M KI. The FE's were calculated using the average of three independent experiments for each electrolyte (replicates shown in **Figure S6**, **Figure S10**, and **Figure S11**). The FE towards H_2 is recorded as a function of potential.

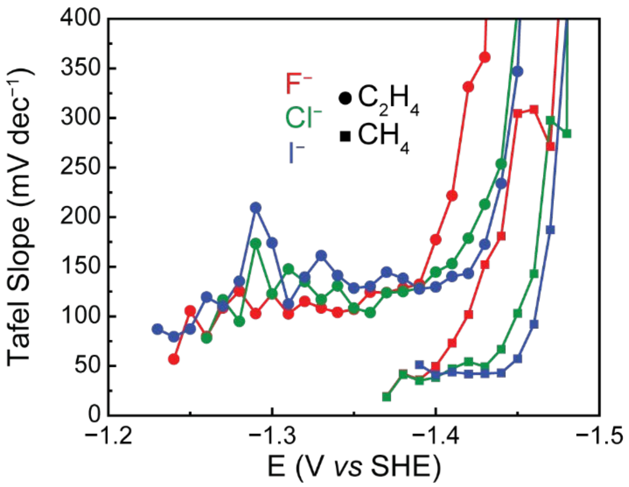

Figure S27: Tafel slope plots for the production rates of ethylene and methane.[9] LSV was performed at a scan rate of 1 mV s^{-1} under CO in the following electrolytes: 0.5 M KF, 0.25 M KF + 0.25 M KCl, and 0.25 M KF +0.25MKI. The reported Tafel slopes were calculated using the average of three independent experiments for each electrolyte (replicates shown in **Figure S6**, **Figure S10**, and **Figure S11**). The potential range used to create the Tafel slope plot was 10 mV.[9]

Table S1: Tafel slopes for C_2H_4 production in various electrolytes.

Electrolyte	Potential Range E (V *vs* SHE)	Tafel Slope (mV dec^{-1})	Tafel Slope Fit (R^2)
0.5 M KF	−1.24 to −1.39	111.5	0.99
0.25 M KF + 0.25 M KCl	−1.26 to −1.39	122.7	1.00
0.25 M KF + 0.25 M KI	−1.23 to −1.40	139.3	1.00

LSV was performed at a scan rate of 1 mV s^{-1} under CO in the presence of various anions. **Figure S26** displays the Tafel slope plot that was used to select the appropriate potential region for calculating the reported Tafel slopes. The reported Tafel slopes were calculated using the average of three independent experiments for each electrolyte (replicates shown in **Figure S6**, **Figure S10**, and **Figure S11**).

Table S2: Tafel Slopes for CH_4 production in various electrolytes.

Electrolyte	Potential Range E (V *vs* SHE)	Tafel Slope (mV dec^{-1})	Tafel Slope Fit (R^2)
0.5 M KF	−1.37 to −1.40	38.7	0.99
0.25 M KF + 0.25 M KCl	−1.38 to −1.43	45.1	0.99
0.25 M KF + 0.25 M KI	−1.39 to −1.44	42.3	1.00

LSV was performed at a scan rate of 1 mV s^{-1} under CO in the presence of various anions. **Figure S26** displays the Tafel slope plot that was used to select the appropriate potential region for calculating the reported Tafel slopes. The reported Tafel slopes were calculated using the average of three independent experiments for each electrolyte (replicates shown in **Figure S6**, **Figure S10**, and **Figure S11**).

Mechanistic Discussion

It is generally assumed that ethylene formation takes place through the rate-limiting electron-mediated dimerization of two CO molecules to form a charged intermediate.[11–13] This is indeed supported by our measured Tafel slope for ethylene formation (112 mV dec^{-1} for KF electrolyte). Under this mechanism, the rate of ethylene formation is dominated by the concentration of *CO on the electrode surface, as well as the rate of C–C bond formation. Methane formation is believed to take place through the formation of a *CHO or *COH intermediate through the hydrogenation of adsorbed CO*.[12,14,15] H_2 can be generated either through the surface reaction between two adsorbed H* or by the direct transfer of H from H_2O in the solution to adsorbed H*.[10,16]

Table S3: H_2 calibration curves for each electrolyte. Details on the calibration can be found in **1.2 Calibration of in-situ EC-MS**.

Electrolyte	Calibration Curve Slope (A · mA^{-1})
0.5 M KF	y = 1.16E−08x
0.25 M KF + 0.25 M KCl	y = 9.98E−09x
0.25 M KF + 0.25 M KI	y = 9.03E−09x

Table S4: Bulk pH values for each electrolyte.

Electrolyte	pH
0.5 M KF	9.82
0.25 M KF + 0.25 M KCl	9.26
0.25 M KF + 0.25 M KI	9.17

Table S5: Bulk pH values for each electrolyte after steady state electrolysis.

Electrolyte	E (V *vs* SHE)	pH
0.5 M KF	−1.1	12.02
	−1.3	12.06
	−1.5	12.46
0.25 M KF + 0.25 M KCl	−1.1	12.09
	−1.3	12.05
	−1.5	12.45
0.25 M KF + 0.25 M KI	−1.1	12.07
	−1.3	12.04
	−1.5	12.41

5. Molecular Dynamics Simulations

Simulation approach and results

Overview of simulations: Classical molecular dynamics (MD) simulations were performed to model 0.5 M potassium iodide (KI) and potassium fluoride (KF) solutions confined between copper electrodes under an applied potential. Adsorbed carbon monoxide was not modeled due to the uncertain arrangement and coverage of adsorbed species, as well as challenges in representing adsorbate polarizability. Force field parameters were carefully selected and validated as described in the sections below. The electrode system was prepared by placing two Cu(100) slabs at a fixed distance from each other with the ion solution confined between them (**Figure S28**). Equal and opposite constant charge densities were applied to the atoms in the top layers of each copper slab to apply a potential across the ion solution. The equilibrium structure of the solvent environment near the negatively charged electrode was then analyzed. We first present key results to support the experimental analysis in the main text. Details on force field selection, validation, system preparation, and analysis are provided in the sections below.

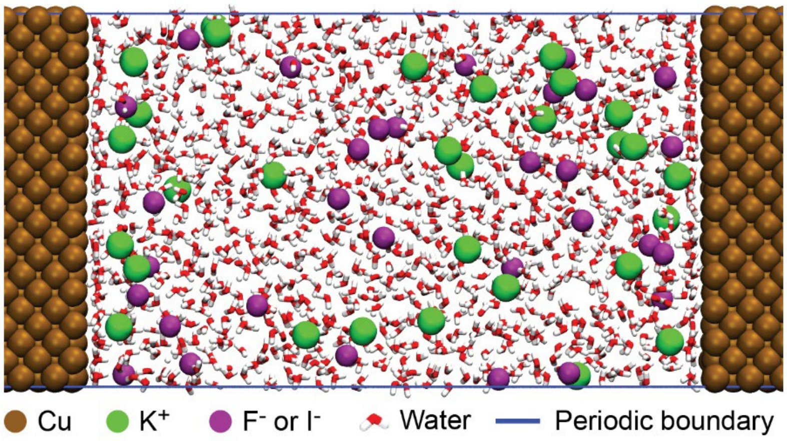

Figure S28: Snapshot of an example simulation system. An ion solution (in this case, 0.5 M KF) is confined between two Cu(100) electrodes. A single periodic image of the system is shown, although all atoms interact across the periodic boundaries. Some water molecules are removed for visual clarity.

Production simulations and analysis: We briefly summarize the methods relevant to the final production simulations in this section; complete methodological details are provided in “Detailed simulation methods” section below. MD simulations of a double-electrode system with either a 0.5 M KF or 0.5 M KI electrolyte solution were performed using the constant-charge method (CCM) to model the applied potential.[17] The charge densities of the electrodes were set such that the negative electrode was at −0.7 V *vs* the potential of zero charge (PZC) to approximate the experimental conditions in the main text.[18,19] Based on CCM calibration simulations, −0.7 V *vs* PZC corresponded to a charge density of 0.0315 C/atom for the electrode surface in the KF system and 0.0300 C/atom for the electrode surface in the KI system.

Simulations were performed using the OPC3 force field for water,[20] Joung-Cheatham parameters for ions,[21] and INTERFACE parameters for the Cu(100) electrodes.[22] Verlet lists were generated with a 1.2 nm cutoff. Van der Waals interactions were modeled using the Lennard-Jones potential with a 1.2 nm cutoff, forces shifted to zero between 1.0 and 1.2 nm, Lorentz-Berthelot combination rules, and a long-range dispersion correction for energy and pressure. Electrostatic interactions were modeled using the smooth particle-mesh Ewald summation method with a 1.2 nm cutoff, 0.12 nm grid spacing, 4th order interpolation, and slab correction.[23] Bonds with hydrogen atoms were constrained using the LINCS algorithm. Production simulations were performed in the *NVT* ensemble for 100 ns with simulation configurations output every 2 ps. Initial configurations were taken from the simulations performed during the calibration of the CCM method (described below), which served as an initial 30 ns equilibration. The temperature was maintained at 298.15 K using a velocity-rescale thermostat with a time constant of 1.0 ps. Position restraints were placed on all electrode atoms. From the production *NVT* trajectory, the number density of each solution component (cation, anion, or water) was computed using the Gromacs tool *gmx density* by time-averaging densities in 0.02 nm increments along the *z*-axis of the simulation box.

<u>Simulation results:</u> **Figure S28** shows simulation snapshots illustrating the entire simulation system while **Figure S29** shows simulation configurations of the region near the negative electrode for KF and KI; videos showing this region for the first 10 ns of the production trajectory are available as **Supplemental Videos S1** (KF) and **S2** (KI). The simulation snapshots and videos show that there is a single layer of water molecules adsorbed to the electrode surface and cations can insert into this water layer to transiently adsorb to the electrode, but anions are largely excluded from the electrode surface. However, there are clearly more fluoride ions near the negative electrode in the KF system than iodide ions in the KI system. These observations are quantified via analysis of component number densities as described below.

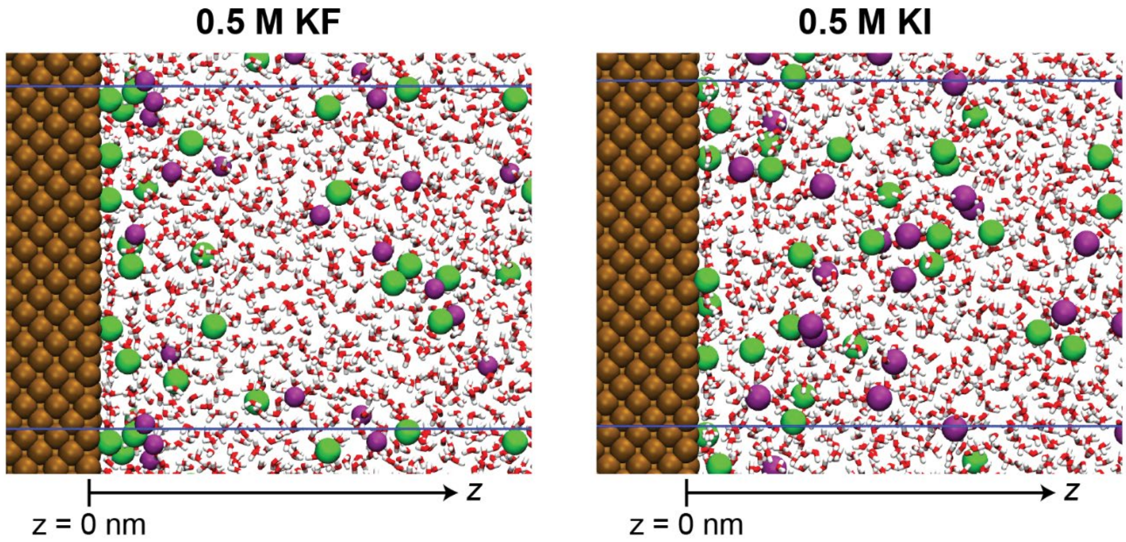

Figure S29: Snapshots of the 0.5 M KF and KI simulation systems focusing on the region near the negative electrode. The *z*-axis is drawn at bottom with the value of z = 0 nm indicated for comparison to **Figure S30**. Multiple periodic images are shown, and some water molecules are removed for visual clarity. The color scheme is identical to the legend in **Figure S28**.

Figure S30a shows the number density of water molecules as a function of the distance along the *z*-axis of the simulation box (*i.e.*, normal to the electrode surface; see **Figure S29**) from the negative electrode. z = 0 nm corresponds to the average position of the top layer of electrode atoms (*i.e.*, those bearing negative partial charges to set the constant surface charge density; see **Figure S29**). Results are compared between simulations of KF (black) and KI (red). Water densities are similar in both solutions and are highly peaked near the surface, indicating a strong affinity of water molecules for the charged surface, as expected. Oscillations in water density near the surface decay over a length scale of approximately 1 nm, which is consistent with the bulk-like behavior of the solution far from the interface.

Figure S30b shows the number density of potassium cations as a function of the distance along the *z*-axis of the simulation. Like water, there is a large peak near the surface in both KF and KI, although the number density of cations is substantially smaller than that of water. This result shows the expected adsorption to the negative electrode; as observed in the simulation videos, adsorption is transient (we note that chemisorption cannot occur in the simulations, so only physisorption is observed). There is substantially more cation adsorption in the KF solution than in the KI solution, which we attribute to the local enrichment of anions as explained below.

Figure **S30c** shows the number density of anions as a function of the distance along the *z*-axis of the simulation. Anion densities are shifted to larger values of z (*i.e.*, farther from the surface) compared to cations due to like-charge repulsion from the negative electrode. These results indicate that there is negligible adsorption of anions to

the surface. Of the two anion species, there is a substantial increase in the concentration of F^- ions closer to the interface compared to I^- ions, with the peak in F^- density coinciding with the region depleted of water density. While the I^- density near the interface is similar to the density in the bulk, this result indicates that F^- ions instead preferentially approach the negative electrode despite like-charge repulsion.

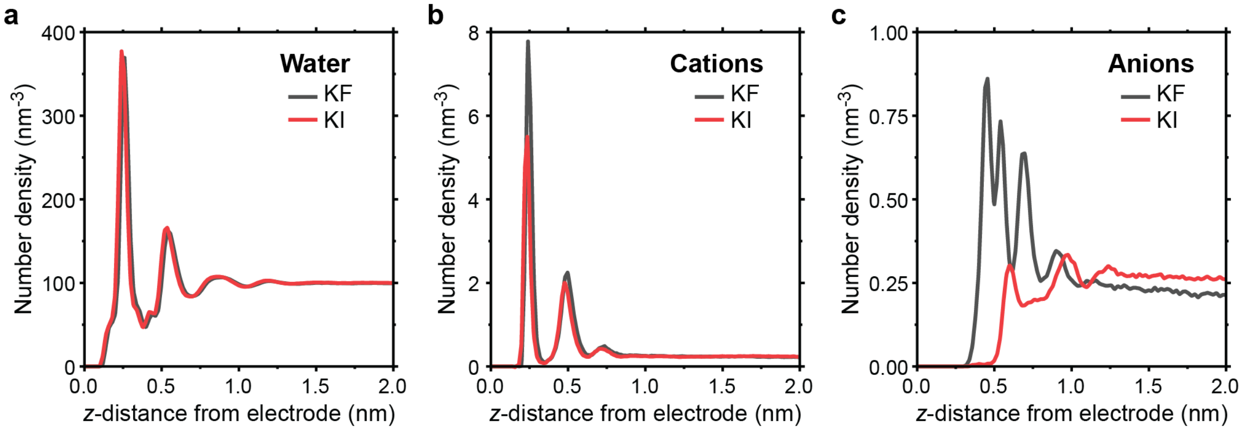

Figure S30: Number densities of (**a**) water, (**b**) potassium cations, and (**c**) fluoride or iodide anions. The *z*-distance is computed as the distance normal to the electrode surface, with *z* = 0 nm corresponding to the surface of the negative electrode (see **Figure S29**).

Finally, we also calculated the electric field as a function of the distance along the *z*-axis of the simulation to determine the extent to which changes in local ion densities impact this field. The electric field was computed by integrating the charge density twice according to Equation 10:

$$\frac{d\phi}{dz} = -\int_0^z \frac{\rho(z')}{\epsilon_0} dz' \qquad (10)$$

As also discussed in the context of Equation 13 below, $\rho(z')$ is the charge density, including contributions from the electrode, solvent, and ions, in a thin volume centered at z' and ϵ_0 is the permittivity of free space. Numerical integration of Equation 10 was performed using the Gromacs tool *gmx potential* with time-averaged charge densities computed as a function of *z* in discrete increments of 0.025 nm. **Figure S31** shows that the electric field was found to be nearly identical for both KF and KI solutions, indicating that the variations in local ion concentration does not significantly affect the interfacial electric field.

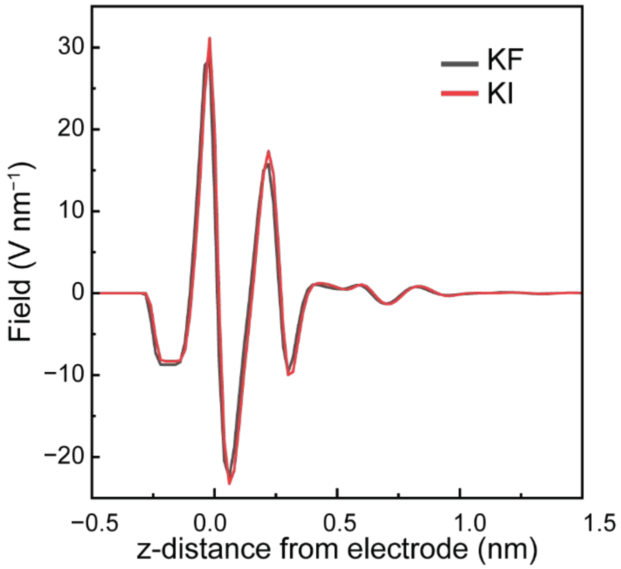

Figure S31: Electric field profile for 0.5 M KF and 0.5 M KI. The z-distance is computed as the distance normal to the electrode surface, with z = 0 nm corresponding to the surface of the negative electrode (see **Figure S29**).

Taken together, the simulation results indicate that in both KI and KF solutions there is a bound water layer near the negative electrode and minimal anion adsorption. However, cations can readily interact with the electrode surface, with a higher concentration of adsorbed cations identified in the KF solution than the KI solution. Fluoride anions are also observed to more closely approach the negative electrode than iodide anions, which may promote the higher cation concentration. In sum, there is a substantial increase in the total concentration of ions near the negative electrode for the KF solution, leading to a distinct solvent environment enriched in ionic species compared to KI. Here, we note that these results are subject to the limitations of the classical MD simulations: no chemisorption can occur, carbon monoxide adsorbates are missing, and only the Cu(100) facet is modeled. Nonetheless, the simulation results reveal differences between KF and KI that support experimental findings pointing to potential anion-mediated differences in behavior at the negative electrode.

Detailed simulation methods

<u>Force field selection and parameters:</u> For aqueous systems, established force fields for water include the rigid three-point TIP3P, SPC, and SPC/E models or the four-point TIP4P family of models, all of which are compatible with common force fields.[24] However, newly developed water models offer substantial improvements in model accuracy when predicting water properties, such as the dielectric constant and bulk water structure, that could influence the behavior observed near a charged electrode. Consequently, we chose to model water using the three-point Optimal Point Charge (OPC3) model which was recently parameterized by searching for globally optimal parameters for a three-point model.[20] OPC3 has been shown to reproduce a wide range of liquid water properties

more accurately than other three-point models like TIP3P and SPC and yields the best overall agreement with experimental values among three-point water models.[20,24] We chose the Joung-Cheatham (J-C) parameters for ions because they have been shown to be accurate for alkali and halide species, including fluoride and iodide.[21] J-C ion parameters differ depending upon on the choice of water model; the developers of OPC3 have suggested that the J-C ion parameter set for the TIP3P force field can be used with the OPC3 model, as validated below.[20] Copper (Cu) was modeled using 12-6 Lennard-Jones parameters from the INTERFACE force field, which was developed to reproduce metal properties relevant to this study (water interfacial energies, adsorbate interactions) using classical Lennard-Jones potentials.[22,25] While we are unaware of prior simulations using the INTERFACE force field with the OPC3 water model, our tests of the interfacial tension at the Cu(111)-water interface revealed that this combination reasonably reproduces experimental values (see below).

All MD simulations were performed using Gromacs 2021.5 using a leapfrog integrator with a 2-fs timestep. A consistent set of Lennard-Jones and electrostatics parameters was adopted for the combination of the OPC3 water model, J-C ion parameters, and INTERFACE force field, with choices validated as described in the next section. Lennard-Jones interactions were modeled with a 1.2 nm cutoff and Lorentz-Berthelot combination rules as suggested for the J-C ion parameters; INTERFACE is also compatible with Lorentz-Berthelot combination rules. Forces were smoothly shifted to zero from 1.0 nm to 1.2 nm using the “force-switch” modifier implemented in Gromacs 2021.5. A long-range dispersion correction for the energy and pressure was applied. Electrostatic interactions were calculated using the smooth particle-mesh Ewald method with a short-range cutoff of 1.2 nm, a grid spacing of 0.12 nm, and fourth-order interpolation. For the final electrode systems (detailed below), the slab correction was applied to produce pseudo-2D Ewald summations.[23,26] Bonds with hydrogen atoms were constrained using the LINCS algorithm.[27] Verlet lists were generated every 20 timesteps using a 1.2 nm neighbor list cutoff.

<u>Force field validation:</u> To the best of our knowledge, the OPC3/J-C/INTERFACE force field combination is novel, so we sought to validate predictions from this combination as described in each subsection below.

OPC3 validation: The OPC3 water model was originally parameterized using a 0.8 nm Lennard-Jones cutoff (without force switching), the long-range dispersion correction for energy and pressure, and particle-mesh Ewald electrostatics. To confirm that the longer Lennard-Jones cutoff used in this work produces reasonable results, we simulated a bulk solution of OPC3 water molecules to calculate experimentally measurable quantities relevant to the present work. A simulation box with initial dimensions of 3 nm × 3 nm × 3 nm was prepared and filled with 811 OPC3 water molecules. Energy minimization was performed using the steepest descent algorithm until the maximum force between atoms was < 1000 kJ mol^{-1} nm^{-2}. The system was then equilibrated at constant *NVT* for 10 ps then equilibrated at constant *NPT* for 30 ps. During equilibration, the temperature was

maintained at 298.15 K using a velocity-rescale thermostat with a time constant of 1.0 ps and the pressure was maintained during *NPT* equilibration at 1 bar using an isotropic stochastic cell rescaling barostat with a time constant of 5.0 ps and compressibility of 4.5 × 10^{-5} bar^{-1}. Production *NPT* simulations were performed using the same thermostat parameters and using a Parrinello-Rahman barostat with a time constant of 5.0 ps and a compressibility of 4.5 × 10^{-5} bar^{-1}. Production simulations were performed for 60 ns with the first 10 ns discarded as additional equilibration. Simulation configurations were output every 2 ps to ensure sufficient sampling.

Using the force field parameters reported in the preceding section, the average density computed from the production simulation was 0.996 ± 0.013 g cm^{-3} which agrees with the literature value of 0.996 g cm^{-3} reported for the OPC3 force field.[20] This value is essentially identical to the experimental density of 0.997 g cm^{-3} for water at 298.15 K and 1 bar.[20] We note that the long-range dispersion correction is important to this result; the density obtained without the dispersion correction was 0.988 g cm^{-3}. The dielectric constant, ε, was computed from fluctuations in the total system dipole moment via Equation 11:

$$\epsilon = \frac{4\,\pi}{3\,k_B T \langle V \rangle}\left(\langle \boldsymbol{M}^2 \rangle - \langle \boldsymbol{M} \rangle^2\right) + 1 \tag{11}$$

In Equation 11, k_B is the Boltzmann constant, T=298.15 K is the temperature, V is the volume of the simulation box during the *NPT* simulation, and $\boldsymbol{M}$ is the total system dipole moment, which is a vector quantity. Angular brackets indicate time-averaged quantities. Dipole fluctuations were computed using the Gromacs tool *gmx dipoles*. The dielectric constant calculated via Equation 11 was 78.7, which is again nearly identical to the literature value of 78.4 ± 1 for OPC3 and 78.4 for experiments.[20] The individual dipole moment of each OPC3 molecule was 2.43 D, again identical to the literature value,[20] and similar to the experimental range of 2.5-3.0 D. We conclude that that the OPC3 model closely matches experimental measurements relevant to this study even with the longer Lennard-Jones cutoff used in our simulations.

OPC3/J-C validation: The OPC3/J-C force field combination was tested by computing the radius of the first solvation shell as an experimentally observable metric relevant to ion solvation. The radius of the first solvation shell was defined as the distance to the first peak of the radial distribution function (RDF) computed between a single ion and surrounding water molecules. Following the procedure of the initial J-C model authors,[21] we placed a single ion (K^+, F^-, or I^-) in a 3 nm × 3 nm × 3 nm simulation box with 811 OPC3 water molecules. Each of the ion-water systems was energy minimized and equilibrated following the same procedure and parameters used for the bulk OPC3 water system. We used the force field parameters described in the preceding section (Lennard-Jones cutoff of 1.2 nm with forces switched from 1.0-1.2 nm, PME with a cutoff of 1.2 nm, and a long-range dispersion correction) which differ from the literature J-C parameters (Lennard-Jones cutoff of 0.9 nm, no force switching, and no long-range dispersion correction).[21] Production *NPT* simulations were then performed for each ion-water

simulation for 2 ns using the same parameters as the OPC3 *NPT* production simulations but with simulation configurations output every 0.5 ps for sufficient sampling.

Using the production simulation trajectories, radial distribution functions (RDFs) were computed using the Gromacs tool *gmx rdf* with a bin size of 0.001 nm. The single ion in each simulation system was used as the reference atom and radial distances were computed to the oxygen atoms of all water molecules. From the resulting RDFs, RDF values within ±0.01 nm of the RDF maximum were fit to a quadratic function. The distance corresponding to the maximum of this function was defined as the radius of the first solvation shell. The radius of the first solvation shell was found to be 0.276 nm for K^+, 0.350 nm for I^-, and 0.262 nm for F^- when using OPC3 water (with TIP3P J-C ion parameters). These values are very similar to literature values for TIP3P water (0.275 nm, 0.351 nm, and 0.263 nm, respectively) and reported experimental values (0.278 nm for K^+, 0.365 nm for I^-, and 0.263 nm for F^-).[21] We conclude that the combination of OPC3 and J-C ion parameters with the longer Lennard-Jones cutoff reproduces the solvation structure of ions in agreement with past reports.

INTERFACE/OPC3 validation: The INTERFACE/OPC3 force field combination was tested by computing the interfacial tension at the copper-water interface. The interfacial tension was computed following the procedure by Heinz *et al* which uses three separate *NVT* simulations to compute the energy of the solid-liquid interface (E_{SL}), liquid-vapor interface (E_L), and solid-vapor interface (E_S).[22] The metal-water interfacial tension is then defined by Equation 12:

$$\gamma_{SL} = \frac{E_{SL} - E_L - E_S}{2A} - T\frac{S_{SL} - S_L - S_S}{2A} \tag{12}$$

A is the surface area of the interface. The second term is an entropic contribution to the interfacial tension that is estimated as 0.06 J m^{-2} following the same assumption as prior work.[22] The interfacial tension was computed for the Cu(111) surface to compare to experimental values. A Cu(111) slab with initial dimensions of 4.09 nm × 4.43 nm × 3.13 nm, totaling 4800 Cu atoms, was created using the Nanomaterial Modeler module in CHARMM-GUI,[28] and solvated with 1,995 OPC3 water models (corresponding to a water layer thickness of approximately 4 nm). The system was equilibrated at constant *NPT* for 100 ps with a semi-isotropic stochastic cell rescale barostat to maintain a pressure of −1.0 bar in the *xy* plane (corresponding to the plane spanned by the copper slab) and 1.0 bar in the *z* direction. The surface area exhibited minimal change during this equilibration as expected.

Systems with the same surface area and either only the copper slab (by removing the water) or only the water (by removing the copper) were then created, and all three systems (water and slab, water only, and slab only) were simulated separately in the *NVT* ensemble for 500 ps. Total system energies were then time-averaged for each of these systems, resulting in E_{SL} = −1378.3 × 10^{-18} J, E_L = −138.3 × 10^{-18} J, and E_S = −1294.3 × 10^{-18} J. Substituting these values into Equation 12 with A = 18.2 × 10^{-18} m^2 and setting

the entropic contribution to 0.06 J m^{-2} yields γ_{SL}= 1.55 J m^{-2}, which is comparable to the previously reported result for the INTERFACE force field using SPC water (1.47 J m^{-2}) and slightly closer to the experimentally measured interfacial tension at 298.15 K (1.70 J m^{-2}).[22] We conclude that the INTERFACE/OPC3 combination is reasonable and slightly more accurate than the common INTERFACE/SPC combination.

Electrode system preparation: Having validated force field parameters, a double-electrode system was constructed to model the distribution of ions and solvent molecules in the presence of an applied potential. Each electrode was modeled as a planar Cu(100) slab because polycrystalline Cu is reported to be dominated by this facet.[29] System preparation was divided into multiple steps (summarized in **Figure S32**) to ensure that the density of the electrolyte solution between the electrodes reached values similar to bulk solution.

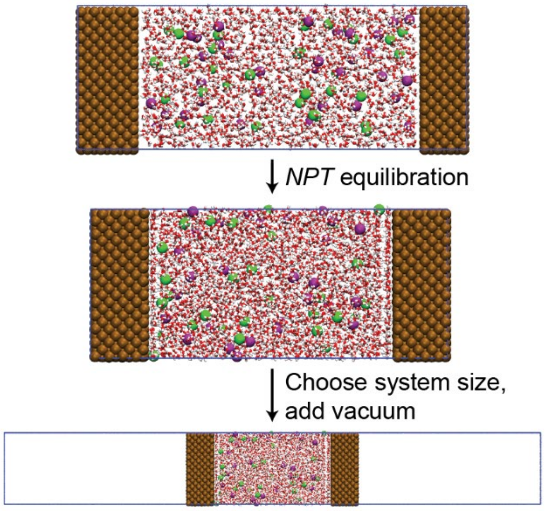

Figure S32: Summary of steps involved in electrode system preparation, illustrated for the KI system. In the first two images, the electrode material interacts with itself across the periodic boundary and hence acts as a single thick slab; adding vacuum in the last step divides this slab into two separate electrodes.

First, a single thick electrode slab with dimensions of 3.98 nm × 3.98 nm × 2.9 nm was prepared using the Nanomaterial Modeler module in CHARMM-GUI,[28] and solvated with 3542 OPC3 water models (corresponding to a water layer thickness of approximately 8 nm) and 33 ion pairs (KF or KI). The system was energy minimized and equilibrated in a 100 ps *NVT* simulation, then equilibrated for an additional 50 ns *NPT* simulation using a semi-isotropic stochastic cell rescaling barostat to maintain a pressure of -1.0 bar in the *xy* plane and 1.0 bar in the *z* direction. For these simulations conventional 3D PME calculations were performed. The electrode area remained close to its initial surface area during these simulations since the area was based on the lattice parameter for Cu(100). The *z*-component of the simulation box varied, however, changing the density of water and effective molarity of the solution far from the surface. In addition, interactions between the electrode surface and both water molecules and ions led to variations in the water

and ion density as a function of the distance from the surface, motivating selection of an appropriate box *z*-dimension and ion concentration prior to further *NVT* simulation.

To choose box dimensions and ion concentrations, we performed an iterative procedure to obtain an ~0.5 M ion solution far from the electrode surface, where the solution should behave as if it were in the bulk. First, a configuration was extracted from the *NPT* simulation trajectory and used as the initial configuration for a 10 ns *NVT* simulations. The number densities of water and both ion species were computed as a function of *z* from this *NVT* simulation and were found to plateau far from the electrode surface as expected. The average density of water in the bulk region where the density plateaued was compared to the density of water computed for a bulk 0.5 M ion solution; if the density was too high, a new configuration with a smaller *z* value was extracted from the *NPT* trajectory and a new *NVT* simulation was performed. This process was repeated until the appropriate water density was found. Next, ion densities were computed from the *NVT* trajectory for the final selected *z* value to determine the molarity of the bulk region. Ion pairs were removed and *NVT* simulations were rerun using the same *z* value until the molarity of the bulk region was close to the 0.5 M target. This procedure was performed separately for the KF and KI systems, leading to final values of z = 9.58 nm with 31 ion pairs for KF and z = 9.55 nm with 29 ion pairs for KI. The number of water molecules was not changed during this procedure.

After determining the appropriate *z* value and number of ion pairs, the final double-electrode system was prepared by introducing a vacuum layer to split the copper slab into two slabs of equal thickness. The thickness of the vacuum layer was chosen such that the total system size in the *z*-dimension was 30 nm. Note that this procedure preserved the distance between electrode surfaces at the value of *z* determined in the preceding step. The large thickness of the vacuum slab was to prevent self-interactions between the electrodes/solution across the periodic boundary condition in the *z* direction and to enable use of the slab correction for Ewald summations, which has been shown to yield more accurate electrostatic calculations for systems with slab geometries by representing the system as pseudo-2D.[23,26] **Figure S33** shows the final sizes for both the KF and KI systems.

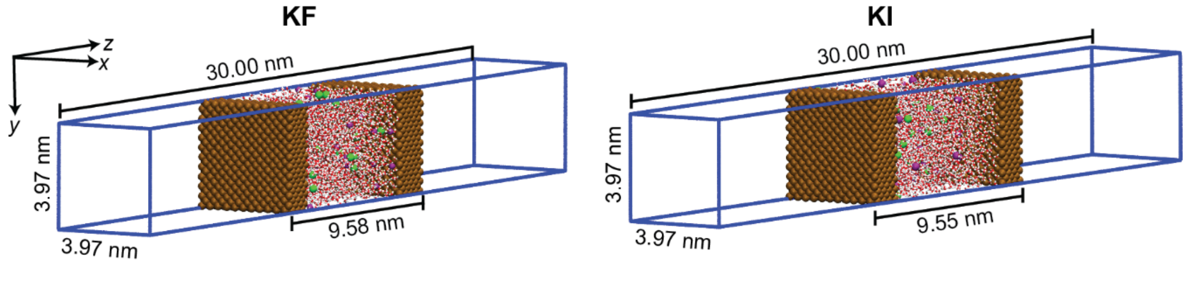

Figure S33: Final system sizes, including the vacuum layer, for the KF and KI systems.

After adding the vacuum layer, the double-electrode system was equilibrated for

10 ns at constant *NVT* using the same force field parameters and options as in prior equilibration steps but with the addition of the Ewald slab correction. In addition, harmonic position restraints were placed on all electrode atoms with a force constant of 400 kJ mol^{-1} nm^{-2} to prevent motion in all three directions. Restraints were applied to prevent disruption of the slab when adding charges to electrode atoms during simulations with an applied potential, and hence were also applied during initial equilibration although no charge was applied to electrode atoms. The final configuration of this *NVT* equilibration was used as the initial configuration for all simulations used to calibrate the constant charge method as discussed in the next section.

<u>Application of potential:</u> We employed the constant charge method (CCM) to model the potential across the electrodes.[17] In this method, the top layer of atoms in each electrode (*i.e.*, those in contact with water) are assigned partial charges that are constant spatially across the electrode surface and invariant with simulation time. This approach corresponds to modeling a spatially uniform, time-invariant charge density at the electrode surface. Each electrode is assigned an equal but opposite charge density to preserve charge neutrality. An alternative approach, called the constant potential method (CPM), instead models an applied potential by permitting the partial charge on each electrode atom to vary as a function of simulation time and in response to the local solvent environment. Prior studies have shown that CCM and CPM produce similar solvation structures at relatively low applied potentials and for dilute electrolyte solutions.[17,30–32] Given the expected similarity of the two methods for the conditions studied in this work, we chose to use CCM to take advantage of its computational efficiency compared to CPM.

The CCM method was implemented in Gromacs by setting the partial charges of atoms in the top layer of each electrode to a constant value (positive for the positive electrode, negative for the negative electrode). Because the CCM method sets charges, and not potentials, the corresponding potential difference between the electrode and bulk solvent is instead computed from equilibrated simulations. Variations in this potential difference as a function of the charge on each electrode atom can be used to construct a calibration curve to permit the selection of a charge needed to reach a target applied potential. We performed separate *NVT* calibration simulations for the KI and KF systems by varying the partial charges on electrode atoms in a series of independent simulations. Each simulation was performed at constant *NVT* for 30 ns, with the first 5 ns excluded as equilibration and simulation configurations output every 2 ps. All force field parameters were the same as in the initial equilibration of the electrode systems, including the application of position restraints to electrode atoms.

The potential was computed as a function of the *z* coordinate of the simulation box (*i.e.*, the axis normal to the electrode surface) using Equation 13:

$$\phi(z) = -\int_0^z \int_0^{z'} \frac{\rho(z'')}{\epsilon_0} dz''dz' \qquad (13)$$

$\rho(z'')$ is the charge density, including contributions from the electrode, solvent, and ions,

in a thin volume centered at z'' and ϵ_0 is the permittivity of free space. Numerical integration of Equation 13 was performed using the Gromacs tool *gmx potential* with time-averaged charge densities computed as a function of *z* in discrete increments of 0.025 nm. While the double integral in Equation 13 is quite sensitive to sampling times, these settings were found to lead to a potential difference less than 0.01 V between the two electrodes when the charge density was set to zero (corresponding to PZC) as expected. Simulations were computed for charge densities ranging from 0 to ±0.030 C/atom for both systems; one additional simulation with ±0.0315 C/atom was performed for KF to reach values of −0.7 V *vs* PZC (see below). For each simulation, the average potential difference between the bulk solution (defined as 14 < *z* 16 nm) and both the negative electrode (*z* < 11 nm) and positive electrode (*z* > 19 nm) was computed as shown in **Figure S34a**. **Figure S34b** shows corresponding relationships (calibration curves) between these potential differences and the charge density of each electrode for both the KI and KF solutions.

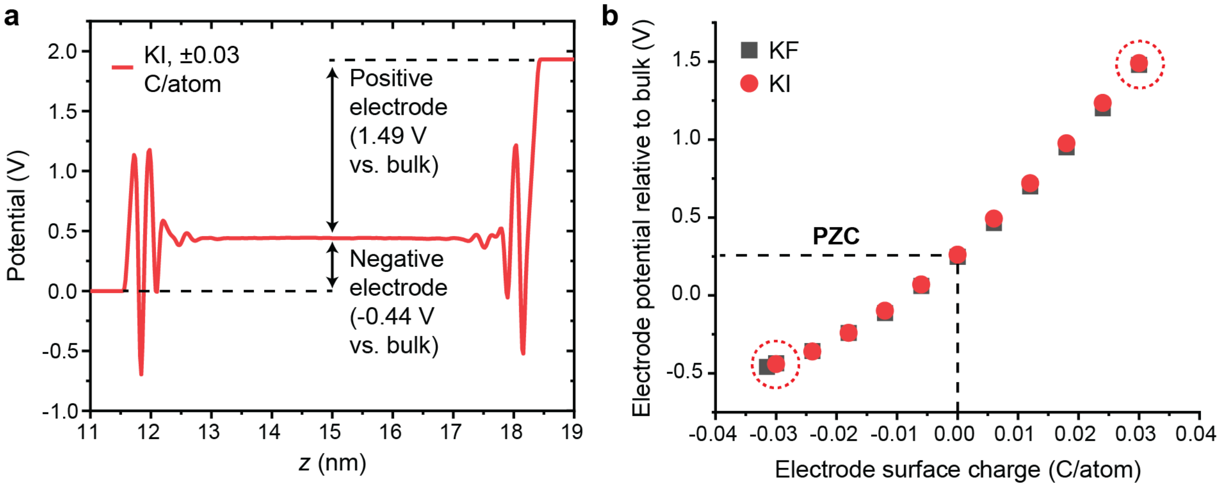

Figure S34: Calibration of constant-charge method (CCM). **(a)** Example calculation of potential vs. *z* value in the simulation box for 0.5 M KI electrolyte with a charge density of ±0.03 C/atom on the electrodes. The region where 14 nm < *z* < 16 nm is defined as the bulk solution and the potential of the negative and positive electrodes is computed relative to the average potential in this region. **(b)** Calibration curve obtained from a series of simulations like those in **(a)** with different electrode surface charge densities. The potential of zero charge (PZC) is equal to the potential of the bulk when the electrodes are both neutral. Dashed circles indicate the value from part **(a)**, which corresponds to approximately −0.7 V *vs* PZC.

The calibration curves were used to inform the selection of electrode charges for the final production simulations. First, we established that the PZC (i.e., the difference in potential between the electrode and bulk solution for neutral electrodes) corresponds to 0.247 V for KF and 0.261 V for KI using these simulation parameters. We estimate that −1.4 V *vs* the standard hydrogen electrode (SHE),[18,19] corresponding to a relevant potential applied in our experiments, is equivalent to −0.7 V *vs.* PZC. Therefore, based

on the calibration simulations, we chose ±0.0315 C/atom (−0.459 V for the negative electrode relative to bulk) for the KF solution and ±0.030 C/atom (−0.441 V for the negative electrode relative to bulk) for the KI solution to model potentials approximately equal to −0.7 V *vs* PZC.

6. References

[1] D. B. Trimarco, S. B. Scott, A. H. Thilsted, J. Y. Pan, T. Pedersen, O. Hansen, I. Chorkendorff, P. C. K. Vesborg, *Electrochim. Acta* **2018**, *268*, 520–530.

[2] D. B. Trimarco, T. Pedersen, O. Hansen, I. Chorkendorff, P. C. K. Vesborg, *Rev. Sci. Instrum.* **2015**, *86*, 075006.

[3] J. X. J. Zhang, K. Hoshino, *Molecular Sensors and Nanodevices*, Academic Press, **2019**, 113–179.

[4] G. Zhang, Z. J. Zhao, D. Cheng, H. Li, J. Yu, Q. Wang, H. Gao, J. Guo, H. Wang, G. A. Ozin, T. Wang, J. Gong, *Nat. Commun.* **2021**, *12*, 5745.

[5] W. Luc, J. Rosen, F. Jiao, Catal. Today **2017**, *288*, 79–84.

[6] K. P. Kuhl, E. R. Cave, D. N. Abram, T. F. Jaramillo, *Energy Environ. Sci*. **2012**, *5*, 7050–7059.

[7] G. Zhang, T. Wang, M. Zhang, L. Li, D. Cheng, S. Zhen, Y. Wang, J. Qin, Z. Zhao, J. Gong, *Nat. Commun*. **2022**, *13*, 7768.

[8] N. Gupta, M. Gattrell, B. MacDougall, *J. Appl. Electrochem.* **2006**, *36*, 161–172.

[9] O. van der Heijden, S. Park, R. E. Vos, J. J. Eggebeen, M. T. M. Koper, *ACS Energy Lett*. **2024**, *9*, 1871–1879.

[10] J. T. Bender, A. S. Petersen, F. C. Østergaard, M. A. Wood, S. M. J. Heffernan, D. J. Milliron, J. Rossmeisl, J. Resasco, *ACS Energy Lett.* **2023**, *8*, 657–665.

[11] K. J. P. Schouten, Y. Kwon, C. J. M. van der Ham, Z. Qin, M. T. M. Koper, *Chem. Sci*. **2011**, *2*, 1902–1909.

[12] J. Li, X. Chang, H. Zhang, A. S. Malkani, M. Cheng, B. Xu, Q. Lu, *Nat. Commun.* **2021**, *12*, 3264.

[13] S. Ringe, E. L. Clark, J. Resasco, A. Walton, B. Seger, A. T. Bell, K. Chan, *Energy Environ. Sci.* **2019**, *12*, 3001–3014.

[14] L. Wang, S. A. Nitopi, E. Bertheussen, M. Orazov, C. G. Morales-Guio, X. Liu, D. C. Higgins, K. Chan, J. K. Nørskov, C. Hahn, T. F. Jaramillo, *ACS Catal*. **2018**, *8*, 7445–7454.

[15] X. Liu, P. Schlexer, J. Xiao, Y. Ji, L. Wang, R. B. Sandberg, M. Tang, K. S. Brown, H. Peng, S. Ringe, C. Hahn, T. F. Jaramillo, J. K. Nørskov, K. Chan, *Nat. Commun.* **2019**, *10*, 32.

[16] H. Chen, J. Iyer, Y. Liu, S. Krebs, F. Deng, A. Jentys, D. J. Searles, M. A. Haider, R. Khare, J. A. Lercher, *J. Am. Chem. Soc*. **2024**, *146*, 13949–13961.

[17] L. Zeng, J. Peng, J. Zhang, X. Tan, X. Ji, S. Li, G. Feng, *J. Chem. Phys.* **2023**, *159*,

091001.

[18] A. Łukomska, J. Sobkowski, *J. Electroanal. Chem.* **2004**, *567*, 95–102.

[19] A. Auer, X. Ding, A.S. Bandarenka, J. Kunze-Liebhäuser, *J. Phys. Chem. C* **2021**, *125*, 5020–5028.

[20] S. Izadi, A. V. Onufriev, *J. Chem. Phys.* **2016**, *145*, 074501.

[21] I. S. Joung, T. E. Cheatham, *J. Phys. Chem. B*. **2008**, *112*, 9020–9041.

[22] H. Heinz, R. A. Vaia, B. L. Farmer, R. R. Naik, *J. Phys. Chem. C* **2008**, *112*, 17281–17290.

[23] I. Yeh, M. L. Berkowitz, *J. Chem. Phys.* **1999**, *111*, 3155–3162.

[24] S. P. Kadaoluwa Pathirannahalage, N. Meftahi, A. Elbourne, A. C. G. Weiss, C. F. McConville, A. Padua, D. A. Winkler, M. Costa Gomes, T. L. Greaves, T. C. Le, Q. A. Besford, A. J. Christofferson, *J. Chem. Inf. Model.* **2021**, *61*, 4521–4536.

[25] K. Kanhaiya, S. Kim, W. Im, H. Heinz, *npj Comput. Mater.* **2021**, *7*, 17.

[26] L. Scalfi, M. Salanne, B. Rotenberg, *Annu. Rev. Phys. Chem.* **2021**, *72*, 189–212.

[27] B. Hess, H. Bekker, H. J. C. Berendsen, J. G. E. M. Fraaije, *J. Comput. Chem.* **1997**, *18*, 1463–1472.

[28] Y. K. Choi, N. R. Kern, S. Kim, K. Kanhaiya, Y. Afshar, S. H. Jeon, S. Jo, B. R. Brooks, J. Lee, E. B. Tadmor, H. Heinz, W. Im, *J. Chem. Theory Comput.* **2022**, *18*, 479–493.

[29] K. J. P. Schouten, Z. Qin, E. P. Gallent, M. T. M. Koper, *J. Am. Chem. Soc.* **2012**, *134*, 9864–9867.

[30] R. Sundararaman, D. Vigil-Fowler, K. Schwarz, *Chem. Rev*. **2022**, *122*, 10651–10674.

[31] D. A. Rakov, J. Sun, P. V. Cherepanov, K. Arano, P. C. Howlett, A. N. Simonov, F. Chen, M. Forsyth, *Energy Environ. Sci.* **2023**, *16*, 3919–3931.

[32] L. Zeng, X. Tan, X. Ji, S. Li, J. Zhang, J. Peng, S. Bi, G. Feng, *Journal of Energy Chemistry* **2024**, *94*, 54–60.
